# Characterizing the Neural Correlates of Response Inhibition and Error Processing in Children With Symptoms of Irritability and/or Attention-Deficit/Hyperactivity Disorder in the ABCD Study®

**DOI:** 10.3389/fpsyt.2022.803891

**Published:** 2022-03-04

**Authors:** Ka Shu Lee, Jingyuan Xiao, Jiajun Luo, Ellen Leibenluft, Zeyan Liew, Wan-Ling Tseng

**Affiliations:** ^1^Department of Experimental Psychology, Medical Sciences Division, University of Oxford, Oxford, United Kingdom; ^2^Yale Child Study Center, Yale School of Medicine, New Haven, CT, United States; ^3^Department of Environmental Health Sciences, Yale School of Public Health, New Haven, CT, United States; ^4^Yale Center for Perinatal, Pediatric and Environmental Epidemiology, Yale School of Public Health, New Haven, CT, United States; ^5^Institute for Population and Precision Health, The University of Chicago, Chicago, IL, United States; ^6^Section on Mood Dysregulation and Neuroscience, Emotion and Development Branch, National Institute of Mental Health, National Institutes of Health, Bethesda, MD, United States

**Keywords:** attention-deficit/hyperactivity disorder, functional magnetic resonance imaging, response inhibition, error processing, irritability, latent class analysis, latent variable modeling

## Abstract

Attention-deficit/hyperactivity disorder (ADHD), characterized by symptoms of inattention and/or hyperactivity and impulsivity, is a neurodevelopmental disorder associated with executive dysfunctions, including response inhibition and error processing. Research has documented a common co-occurrence between ADHD and pediatric irritability. The latter is more characterized by affective symptoms, specifically frequent temper outbursts and low frustration tolerance relative to typically developing peers. Shared and non-shared neural correlates of youths with varied profiles of ADHD and irritability symptoms during childhood remain largely unknown. This study first classified a large sample of youths in the Adolescent Brain Cognitive Development (ABCD) study at baseline into distinct phenotypic groups based on ADHD and irritability symptoms (*N* = 11,748), and then examined shared and non-shared neural correlates of response inhibition and error processing during the Stop Signal Task in a subset of sample with quality neuroimaging data (*N* = 5,948). Latent class analysis (LCA) revealed four phenotypic groups, i.e., high ADHD with co-occurring irritability symptoms (*n* = 787, 6.7%), moderate ADHD with low irritability symptoms (*n* = 901, 7.7%), high irritability with no ADHD symptoms (*n* = 279, 2.4%), and typically developing peers with low ADHD and low irritability symptoms (*n* = 9,781, 83.3%). Latent variable modeling revealed group differences in the neural coactivation network supporting response inhibition in the fronto-parietal regions, but limited differences in error processing across frontal and posterior regions. These neural differences were marked by decreased coactivation in the irritability only group relative to youths with ADHD and co-occurring irritability symptoms and typically developing peers during response inhibition. Together, this study provided initial evidence for differential neural mechanisms of response inhibition associated with ADHD, irritability, and their co-occurrence. Precision medicine attending to individual differences in ADHD and irritability symptoms and the underlying mechanisms are warranted when treating affected children and families.

## Introduction

Attention-deficit/hyperactivity disorder (ADHD), characterized by symptoms of inattention and/or hyperactivity and impulsivity, is a common neurodevelopmental disorder in youths (1). Irritability, the frequent manifestation of temper outbursts and low frustration tolerance compared to peers (2, 3), is also common in child psychiatry. Recent studies suggest that a significant proportion of youths with ADHD also show marked symptoms of irritability or irritability-related emotion dysregulation, with estimates commonly ranging between 30 and 50% (2, 4, 5). Conversely, youths with disruptive mood dysregulation disorder (DMDD), for which severe irritability and temper outbursts are hallmark symptoms, show high rates of co-occurring ADHD (6). Irritability symptoms in ADHD can be particularly impairing and difficult to manage because temper outbursts and extreme frustration exacerbate maladaptive behavior with peers and caregivers and increase the risk of developing aggressive behaviors (5, 7), anxiety, and depression (8). Despite the high prevalence of co-occurring ADHD and irritability, which is associated with greater impairment in social functioning and mental health than either alone, the underlying mechanisms of this co-occurrence remain unclear (9). The goal of this study was to use data-driven latent modeling approaches to identify dissociable neural correlates of ADHD, irritability, and their co-occurrence during the Stop Signal Task (SST), a well-validated cognitive control task (10–12).

A large body of literature demonstrates deficits in executive functions [for a review, see (13)], i.e., top-down cognitive functions exerting control over one's behavior, in youths with ADHD. Symptoms of inattention and hyperactivity/impulsivity have been linked to difficulty monitoring and adjusting one's behavior to environmental demands, potentially due to top-down cognitive dysfunctions (14, 15). Two interlinked but independent neurocognitive processes, response inhibition and error processing, are particularly relevant to behavioral regulation that requires attending to errors and inhibiting maladaptive behaviors (12, 16). Response inhibition refers to the suppression of behavior that is considered erroneous in a given context, while error processing refers to the detection of environmental signals that are inconsistent with certain rules or regularities in a given context (16). Functional Magnetic Resonance Imaging (fMRI) research suggests that youths with ADHD show aberrant neural activation in inferior frontal and temporal/parietal nodes during response inhibition and error processing (10, 17). Some also found altered brain activation associated with ADHD severity in the salience network, such as the anterior insular and anterior cingulate cortices, during tasks similar to the SST that require response inhibition (10, 18). However, it is noteworthy that there is considerable heterogeneity in the statistical procedures, fMRI tasks, and clinical samples recruited in these independent studies, and that the functional brain alterations found might be confounded by co-occurring symptoms (19).

Compared to the large body of literature in ADHD, the neural underpinnings of cognitive control, particularly response inhibition, in irritability have received less attention. A growing body of work using fMRI and functional near infrared spectroscopy (fNIRS) with tasks involving inhibitory and cognitive control suggests that higher irritability symptoms are associated with altered activation in the prefrontal regions (20–22) and regions in the salience network [including the anterior cingulate cortex and amygdala; (22)]. A recent study extended these findings by examining functional connectivity and found functional connectivity within the sensorimotor network, and between sensorimotor and frontoparietal and medial frontal networks to be predictive of irritability severity in a frustrative cognitive flexibility task that requires inhibition (23). Directly relevant to the current study, a large transdiagnostic study used the SST in 320 adolescents (including ADHD) and found that irritable mood was associated with reduced activation in the frontal and temporal cortices during inhibitory control (24).

Despite the emerging literature on the neural correlates of cognitive control in irritability, many past studies have small sample sizes (*N* < 100), and almost none directly investigated the neural correlates underlying the co-occurrence of irritability and ADHD vs. either phenotype presenting alone. One exception is the study by Pagliaccio et al. (25) where they compared the neural correlates of sustained attention on a global-local attention task between youths with ADHD and those with DMDD, the majority (77%) of which also had a lifetime diagnosis of ADHD. The authors reported that youths with DMDD showed aberrant activations in areas such as the right paracentral lobule and superior parietal lobule, while both youths with DMDD and ADHD showed blunted compensatory activation in the frontal and parietal regions. Although the current SST does not specifically probe attention processes *per se*, inhibitory control is partly supported by attention control (18), suggesting the value of examining neural differences associated with inhibitory control functions in youths with ADHD and co-occurring irritability symptoms.

Methodologically, past fMRI studies in youths with ADHD and irritability have focused predominantly on regional, task-dependent neural activation (19). However, a multivariate approach focusing on coactivation and/or functional connectivity among regions may facilitate the discovery of brain-behavior associations and neurocognitive differences [(26, 27), see a review by Cooper et al. (28)]. Therefore, the current study leveraged the large dataset (*N* = 11,875) from the Adolescent Brain Cognitive Development (ABCD) study at baseline (29, 30) to identify differential neural correlates of cognitive control in youths with ADHD, irritability, and the co-occurrence of ADHD and irritability. We focused on the baseline cross-sectional data, instead of longitudinal data, to establish the baseline prevalence of ADHD, irritability, and their co-occurrence, because the baseline data provides the largest possible sample. This lays the foundation to investigate the neural underpinnings that link to future mental health risks and developmental outcomes among the ADHD and/or irritability phenotypes. Specifically, we first used a latent phenotyping approach (i.e., latent class analysis [LCA]) to identify distinct phenotypes of ADHD and/or irritability symptoms across diagnostic categories using the ABCD dataset. We also explored any sex differences in the distinct phenotypes of ADHD and/or irritability, as it has been found that boys are disproportionately more likely to be reported as having ADHD (and irritability symptoms in clinical samples) than girls (31), which warrants validation in a large sample. Next, we used latent variable modeling to derive the neural coactivation networks that support response inhibition and error processing during the SST [e.g., (27, 32)], and tested for any neural differences between the ADHD and/or irritability phenotypes. A conventional univariate, regional approach was adopted as a secondary analysis, allowing for the comparison of results at the regional level. We also conducted a sex by phenotype exploratory analysis on the regional activation results. Informed by the only study that reported blunted neural activation in both ADHD and irritability-related youths (25), we hypothesized that the ADHD and irritability groups would show decreased neural coactivation (less so for regional activation) during response inhibition and error processing on the SST relative to typically developing peers.

## Materials and Methods

### Participants

This study used baseline data from the ABCD study Release 3.0, a population-based study of adolescent brain and cognitive development, which is designed to follow 11,875 demographically diverse youths aged 9–10 years longitudinally for 10 years across 21 research sites in the United States (28, 29). Briefly, the ABCD study comprises physical and mental health assessments and socio-demographic surveys for youths and their caregivers, as well as a battery of neuroimaging tasks examining core cognitive-affective brain functions, including response inhibition and error processing assessed by the SST. We first included *N* = 11,748 youths with complete assessments of irritability and ADHD symptoms from the parent diagnostic interview for the DSM-5 Kiddie Schedule for Affective Disorders and Schizophrenia (K-SADS) to derive distinct phenotypic groups based on ADHD and irritability symptoms using LCA. Our rationale of using the baseline sample of *N* = 11,748 in the LCA was to obtain the largest possible sample, which allowed us to assay a diverse spectrum of ADHD and irritability symptoms, thereby generating more accurate community prevalence estimates of different clinical phenotypes [e.g., ~3% for DMDD (33, 34)] and retaining decent subgroup sample sizes (and thus power) for further testing group differences in neural responses on the SST. Next, we examined shared and non-shared neural correlates of response inhibition and error processing in a subset of the LCA sample with complete and quality neuroimaging data on the SST (*N* = 5,948; see Supplementary Materials for details), following similar procedures as the past modeling studies (27, 35). Of the 5,948 youths, 89% cases provided complete socio-demographic information. Comparisons between the included (*N* = 5,948) and excluded (*N* = 5,930) samples suggest modest but significant differences in all socio-demographic variables (see Supplementary Materials, Supplementary Table 1). Subsequent visual inspection and statistical comparisons also revealed significant group differences in all socio-demographic variables between the typically developing group and clinical phenotypes derived from the LCA (see Supplementary Table 2). We therefore adopted the past studies' approach to include all the socio-demographic variables as covariates in our modeling (27, 35, 36). All data were derived from Release 3.0 and accessed under National Institute of Mental Health Data Archive study ID 9487.

### Measures

#### Socio-Demographics and Clinical Symptoms

##### Socio-Demographics

Child's age was extracted from the parent diagnostic interview for K-SADS. Child's sex, race, ethnicity, caregiver education, caregiver marital status, and total combined family income for the past 12 months were retrieved from the Parent Demographics Survey at baseline.

##### Child Irritability Symptoms

Irritability symptoms were measured using four items across relevant diagnostic categories from the parent-reported K-SADS [(37); also see a review (38)]. These included one item “Irritability Present” from the Major Depressive Disorder module, one item “Temper outbursts occur 3 or more times per week” from the DMDD module, and two items “Often touchy or easily annoyed Present” and “Often loses temper Present” from the Oppositional Defiant Disorder module. These item-level responses on the K-SADS were binary coded (0 = absent; 1 = present). Confirmatory factor analysis supported a coherent one-factor structure in the selected items, [$\chi_{\left(2\right)}^{2}$ = 17.26, *p* < 0.001], Comparative Fit Index (CFI) = 1.00, Tuker-Lewis Index (TLI) = 1.00, Root Mean Square Error of Approximation (RMSEA) = 0.03 [0.02, 0.04], Standardized Root Mean Square Residual (SRMR) = 0.03.

##### Child ADHD Symptoms

ADHD symptoms were measured using 18 items from the ADHD module of the K-SADS. Items included “Difficulty sustaining attention,” “Often makes careless mistakes,” and “Runs or climbs excessively.” Consistent with past research assessing child ADHD via the K-SADS [e.g., (39)], confirmatory factor analysis showed an excellent one-factor structure among the ADHD items, [$\chi_{\left(135\right)}^{2}$ = 3475.58, *p* < 0.001], CFI = 1.00, TLI = 1.00, RMSEA = 0.05 [0.05, 0.05], SRMR = 0.03.

### Neuroimaging Task

The SST was used to index cognitive control-related brain functions and behavioral performance during fMRI in the ABCD study (11, 29, 30, 36). Comparing with the other fMRI tasks and resting state data, the SST probes response inhibition and error processing, two key neurocognitive processes underlying ADHD symptoms (10, 14, 15) and have the potential to shed light on the differential neural mechanisms in ADHD cases with vs. without co-occurring irritability symptoms (25). Suboptimal inhibitory control may contribute to aberrant responses to frustration (40); however, there has been limited empirical effort investigating the functional neural correlates of cognitive control in irritability (40, 41).

In brief, the SST asks participants to indicate the direction of left- or right-facing arrows (“Go” signals) as quickly and accurately as possible, but also not to respond following an upward-pointing arrow that indicated a “Stop” signal. The SST used in the ABCD study was in an event-related design comprised of two runs, each having 180 trials inclusive of 30 “Stop” trials; hence, there were 150 × 2 = 300 “Go” trials and 30 × 2 = 60 “Stop” trials in total. Each trial lasted 1 s. An algorithm caused the time between the “Go” and “Stop” signals to vary depending on the participant's accuracy in the previous trial, thus achieving an overall success rate of 50%. The behavioral task measure of inhibition ability was Stop Signal reaction time (SSRT). To address experimental issues of SST noted previously (42), we estimated SSRT using the integration method, in which estimates were adjusted for behavioral parameters including successful inhibition rate, “Go” reaction time omissions, and premature responses on “Stop” trials.

### Neuroimaging Measures

**Figure 1:**
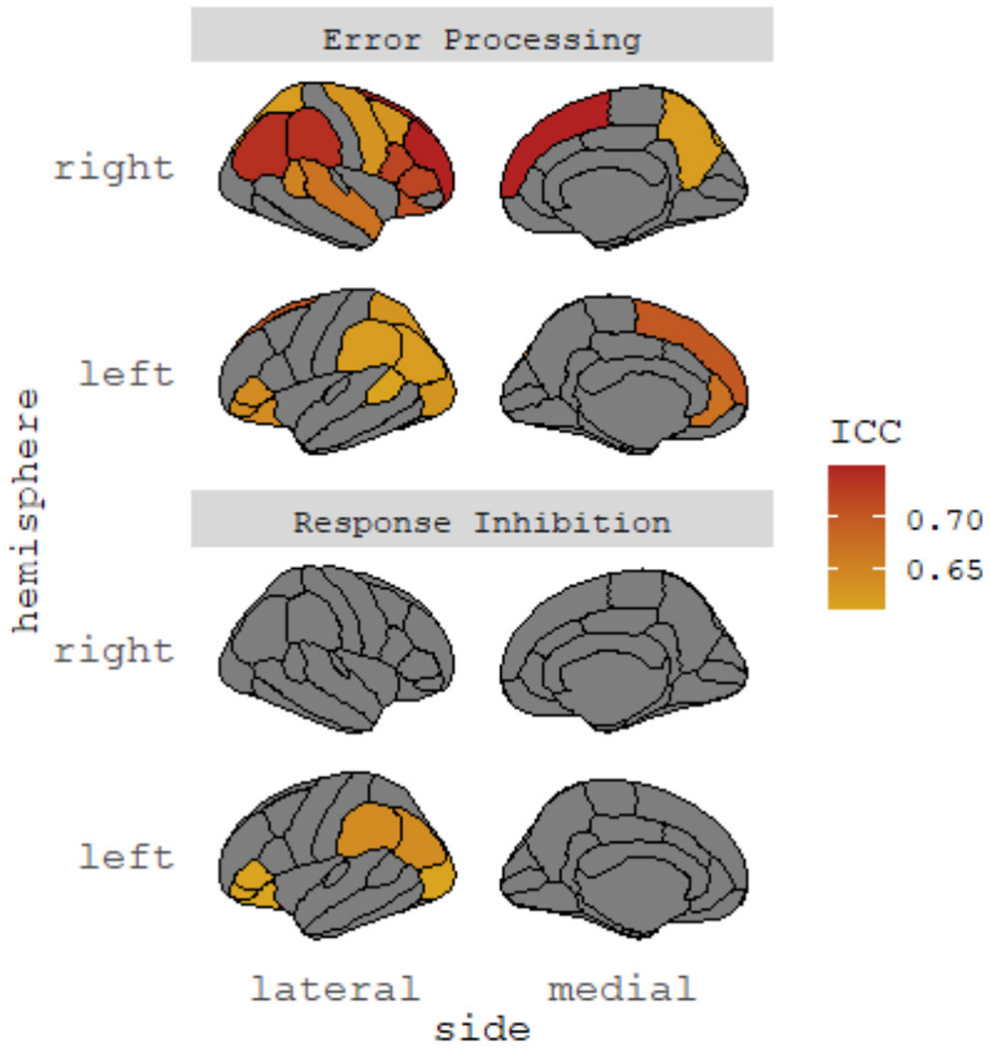
Selected brain regions with high test-retest reliability. ICC, intraclass correlation coefficient. The identified cortical regions showed significant test-retest reliability (ICCs > 0.60) in Korucuoglu et al. (45). No subcortical regions showed significant test-retest reliability in the study. Brain regions identified were cross-checked with Hagler et al. (44).

Task-dependent functional brain activations were indexed by average beta weights. Data were pre-processed by the ABCD Data Analysis and Informatics Center (43). To empirically derive robust neural coactivation networks based on task-dependent regional activation during the SST, we focused on regions with at least moderate test-retest reliability (i.e., intra-class correlation coefficient [ICC] >0.60) based on Korucuoglu et al. (44), given concerns regarding poor test-retest reliability of common task-fMRI measure (45). Korucuoglu et al.'s study was one of the few empirical studies available at the time of data analysis that examined test-retest reliability of the same SST Task used in the ABCD study and rigorously identified the most robust brain regions in a data-driven manner (with at least moderate to high reliability in the sample), which was rare in the literature. We did not select regions of interest from the highly heterogeneous literature as recent meta-analyses suggested a lack of functional neural convergence across neurocognitive domains in youths with ADHD (19) and youths with irritability symptoms (46). However, we acknowledged the limitations (e.g., a small sample of young female adults) of the selection of regions based on Korucuoglu et al.'s study (see Discussion). A total of 6 and 23 regions were selected for response inhibition and error processing, respectively (see Figure 1 and Supplementary Materials, Supplementary Table 3). As noted below, we also conducted a whole-brain analysis inclusive of all available brain regions in the SST dataset.

### Statistical Procedures

Latent variable modeling was conducted in Mplus version 8.3 (47), with models specified using maximum likelihood estimation with robust standard errors. All other statistical procedures, including regional group comparisons, were conducted in R version 4.0.3 (48) with the use of the lme4 package (49). Statistical analyses controlled for all socio-demographic covariates as described previously, with the nesting structure of scan site taken into account because of potential scanner differences between sites as in past studies utilizing the ABCD dataset [e.g., (27, 35, 36)]. Statistical tests were corrected for false-discovery rate (FDR), and significant at alpha = 0.05. Standardized estimates were reported unless otherwise specified.

#### Latent Class Analysis to Identify Latent Phenotypes

LCA empirically derived latent subgroups based on observed irritability and ADHD symptoms at the item-level (4 and 18 items, respectively) as assessed by the K-SADS in the full sample at baseline (*N* = 11,748). A one-class solution was first fitted to the data, followed by successive solutions with one additional class of the previous solution until the best fitting solution was identified. The various solutions were compared based on model fit indexed by the Akaike Information Criterion (AIC), Bayesian Information Criteria (BIC), sample-size adjusted BIC (ABIC), entropy, Vuong-Lo-Mendell-Rubin likelihood ratio test (VLMR LRT), Lo-Mendell-Rubin adjusted likelihood ratio test (LMR adjusted LRT), and parametric bootstrapped likelihood ratio test (PB LRT). Following previous research (31, 35, 50), the best fitting model was chosen based on various criteria, including the greatest relative decreases in AIC, BIC, and ABIC, entropy >0.90, statistically significant LRTs with *p* < 0.05 for all tests, and class proportions >2% of the full sample given the estimated community prevalence of severe child irritability in past research [e.g., (51, 52)]. However, because there is no gold standard for determining the optimal LCA model, comparisons between class solutions must take into account the research questions and prevalence estimates of the clinical phenomenon of interest (31, 50, 53).

#### Latent Variable Modeling to Identify Networks of Response Inhibition and Error Processing

We conceptualized individual brain regions contributing to a coherent factor structure as a neural coactivation network supporting response inhibition and error processing during the SST. Compared to a conventional regional approach, functional neuroimaging measures derived from the latent variable modeling reduce measurement error, which may increase statistical power (28). A three-step procedure was used to derive a latent structure of the SST neuroimaging measures [e.g., (32)]. First, based on the selected brain regions with at least moderate test-retest reliability (i.e., 6 regions for response inhibition and 23 regions for error processing) (44), we ran a series of exploratory factor analyses (EFAs) exploring the best fitting structures from the range of one to three factors for a response inhibition model and an error processing model, respectively. The exploratory factor structures were closely evaluated and discarded using the criteria proposed by Muthén and Muthén (54), namely (i) eigenvalues <1 across all factor structures explored; (ii) presence of cross-loading coefficients >0.10 between two or more indicators; (iii) indicators with factor loading coefficients <0.30; and (iv) number of indicators per each factor identified ≤2. Statistical significance of the factor loadings was not used to determine the suitability of a factor solution given that the large sample size rendered even the most subtle effects statistically significant. Second, the most suitable factor structures (response inhibition and error processing, separately) identified in the EFAs were then replicated in the context of confirmatory factor analysis to allow for subsequent measurement invariance testing and multi-group comparisons of neural coactivation patterns. Similarly, we replied on model fit indices, but not statistical significance, to evaluate the applicability of the identified neural coactivation networks. Given that large sample sizes often bias the chi-square tests, model fit was indexed by CFI, TLI, RMSEA, and SRMR. Following published guidelines, a model with CFI and TLI ≥ 0.95, RMSEA ≤ 0.05, and SRMR ≤ 0.08, are considered a good fit with the observed data (55–57), while a model with CFI and TLI ≥ 0.95, and RMSEA ≤ 0.10 indicates adequate fit (56, 57). Third, to ensure that the identified factor structures applied to the four latent classes of youths identified based on child irritability and ADHD symptoms, we then conducted measurement invariance testing using latent class membership as a grouping variable. A conventional bottom-up approach was used to test for measurement invariance of the configural model, metric model, and finally, the scalar model across latent classes (27, 58). Again, given the large sample size, we adopted the criteria proposed by Chen (59) to compare fit indices of the three models, rejecting measurement invariance when ΔCFI > 0.01 and ΔRMSEA > 0.015.

#### Differences in Neural Correlates Across Latent Phenotypes

Models achieving measurement invariance were then used to test for significant group differences in latent intercepts in the context of multi-group confirmatory factor analysis via the model constraint procedures in Mplus. These group comparisons were conducted separately for the response inhibition and error processing models. Finally, as a secondary region-level analysis, a series of linear mixed models tested for group differences in each of the constituent brain regions of the response inhibition coactivation network and the error processing coactivation network. All multiple group comparisons were FDR-corrected. While there is still ongoing discussion regarding the computation of effect sizes for specific effects derived from complex latent variable and multi-level modeling procedures [e.g., (60)], we performed *post-hoc* calculations of Cohen's *d* (61, 62) and eta-squared (61, 63) as the effect size proxies of the estimates for the group differences in latent and regional neural differences on the SST. The effect size interpretation for Cohen's *d* is 0.2, 0.5, and 0.8 for small, medium, and large effects, respectively; and the interpretation for eta-squared is 0.01, 0.06, and 0.14 for small, medium, and large effects, respectively.

#### Differences in Behavioral Performance Across Latent Phenotypes

A linear mixed model compared behavioral performance on the SST (i.e., Stop Signal reaction time) across latent classes.

### Quality Control, Motion Effects, and Outliers

We included various quality control parameters to screen out cases with potentially problematic neuroimaging data (29, 35, 36). These included the presence of head motion ≥0.9 mm average frame-wise displacement on the SST, degrees of freedom across runs ≤200, and poor data quality indicted by FreeSurfer's quality control.

Methodological and programming issues regarding the SST in the ABCD study were acknowledged and addressed in the current study (42, 64). Briefly, there were task coding errors in the measurement of Stop Signal Delay (SSD), resulting in task performance measurement errors and data coding issues (42). After consultation with the ABCD Data Analysis and Informatics Center, we decided to screen out cases based on the following criteria: (i) cases that did not pass the SST behavioral performance check (indexed by the SST performance flag); (ii) cases with the unresolved task coding error wherein a speedy response <50 ms was made when SSD was 50 ms (42); and (iii) cases where the number of Stop trials with 0 ms SSD was ≥10%. Note that several programming issues (42) were still under investigation when the current study was conducted (64).

Finally, inspection of the SST neuroimaging data suggested that ~25% of brain regions showed extreme outliers, defined as ±3 standard deviations and skewness >2 of the average beta weight of that brain region (27, 35). We used winsorization to harmonize the extreme data points [e.g., (35, 65)], i.e., an average of 0.93% (max. 2.34%) individual data points across the SST regions for each participant. Taken together, these quality control procedures allowed for a balance between generalizability and a conservative approach that analyzes the best quality cases only (66, 67).

### Supplementary Analyses

Four sets of supplementary analyses were conducted.

#### Latent Variable Modeling Using All Available Brain Regions

Since Korucuoglu et al. (44) studied a sample of young adults (age range = 21–24 y), the reliable brain regions found in that study might not reflect those in our adolescent sample. We therefore supplemented our main modeling results using all 34 bilateral sets of brain regions for response inhibition and error processing on the SST, respectively (43) without pre-selecting regions for reliability. The same model selection procedures (i.e., exploratory factor analysis followed by confirmatory factor analysis and then measurement invariance testing) were applied to the supplementary modeling.

#### Potential Sex by Latent Phenotype Interactions

Taking advantage of the large sample, we also explored sex by latent class interactions in each of the constituent brain regions of the response inhibition and error processing coactivation networks. Because the comparison of latent variables within a multiple hierarchical structure is not available in the current version of Mplus, the analyses were performed at the regional level.

#### Group Comparison With Family Clustering

We also conducted *post-hoc* group comparisons using family as a clustering variable in Mplus to account for the interdependent, nested data within family. The same modeling procedures were used for the group comparisons using a priori selected regions and all available brain regions on the SST.

#### Group Comparison Removing All Covariates

Given that various socio-demographic differences between the LCA groups were of small to medium effect sizes (see Supplementary Table 2), we re-ran the latent group comparisons with all covariates removed on a *post-hoc* basis.

## Results

### Latent Class Membership and Sample Characteristics

**Figure 2:**
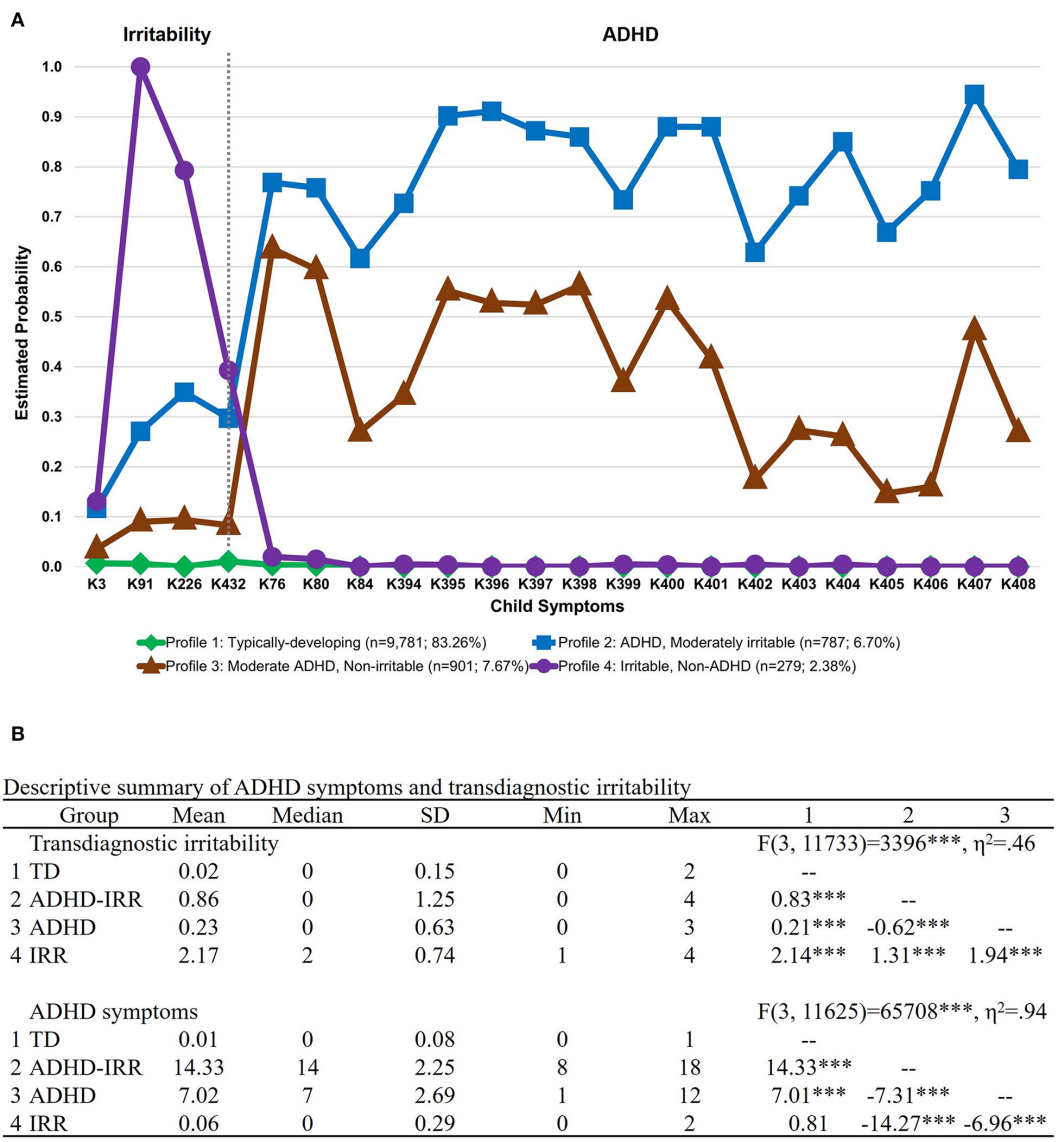
Latent class membership based on ADHD symptoms and transdiagnostic irritability. Symptoms were derived from item-level symptoms assessed by the parent interview of the DSM-5 Kiddie Schedule for Affective Disorders and Schizophrenia (K-SADS). **(A)** Latent class graph visualizing the estimated probability of ADHD symptoms and transdiagnostic irritability for each group. **(B)** Descriptive summary of ADHD symptoms and transdiagnostic irritability for each latent class group based on item-level symptom count on the K-SADS. The numbered columns represent adjusted pairwise comparisons following the omnibus test of differences in symptom counts across groups. ADHD, attention-deficit/hyperactivity disorder; IRR, irritability; TD, typically developing. ***p < 0.001.

Figure 2A depicts the LCA results that classified youths based on child irritability and ADHD symptoms at the item level on the K-SADS in the full sample at baseline (*N* = 11,748). A four-class solution was identified as the best fitting solution, AIC = 56111.24, BIC = 56782.04, ABIC = 56492.85, entropy =0.97, VLMR LRT *p* < 0.001, LMR adjusted LRT *p* < 0.001, PB LRT *p* < 0.001, smallest class proportion = 2.4%. Compared to the five-class solution, there was a drop in statistical significance in the PB LRT, suggesting that the four-class solution was superior (see Table 1). As depicted in Figure 2A, the four latent classes were: (1) typically developing youths with low irritability and low ADHD symptoms (*n* = 9,781, 83.3%); (2) youths with high ADHD symptoms and co-occurring irritability (*n* = 787, 6.7%); (3) youths with moderate ADHD symptoms and low irritability (*n* = 901, 7.7%); and (4) youths with high irritability and low ADHD symptoms (*n* = 279, 2.4%). To provide a more concrete overview of the differences in symptom profiles, Figure 2B presents a descriptive summary of the ADHD and irritability symptoms based on item-level symptom counts on the K-SADS in each LCA group. The summary showed that while the ADHD + irritability phenotype has the highest number of ADHD symptoms amongst all groups (mean = 14.33, SD = 2.25), their level of co-occurring irritability symptoms (mean = 0.86, SD = 1.25) is much lower, although variability was greater, than that of youths with high irritability symptoms only (mean = 2.17, SD = 0.74), *p*s < 0.001. Youths with moderate ADHD symptoms (mean = 7.02, SD = 2.69) also showed a mild level of irritability symptoms (mean = 0.23, SD = 0.63) relative to typically developing peers (mean = 0.02, SD = 0.15), *p* < 0.001.

**Table 1:** Model fit information of latent class analysis.

	**AIC**	**ΔAIC**	**BIC**	**ΔBIC**	**ABIC**	**Entropy**	**VLMR LRT**	**LMR adjusted LRT**	**PB LRT**	**Best log likelihood value replicated?**	**Class proportions > 2%?**
1 Class	121829.67	–	121991.85	–	121921.93	–	–	–	–	–	–
2 Classes	61798.18	−60031.50	62129.89	−59861.95	61986.89	0.97	*p* < 0.001	*p* < 0.001	*p* < 0.001	Yes	Yes
3 Classes	57831.99	−3966.19	58333.24	−3796.65	58117.15	0.96	*p* < 0.001	*p* < 0.001	*p* < 0.001	Yes	Yes
**4 Classes**	**56111.24**	**−1720.75**	**56782.04**	**−1551.20**	**56492.85**	**0.97**	***p*** **< 0.001**	***p*** **< 0.001**	***p*** **< 0.001**	**Yes**	**Yes**
5 Classes	55154.14	−957.10	55994.48	−787.56	55632.20	0.96	*p* = 0.0056	*p* = 0.0057	*p* < 0.001	Yes	Yes

*AIC, Akaike Information Criterion; BIC, Bayesian Information Criteria; ABIC, sample-size adjusted BIC; VLMR LRT, Vuong-Lo-Mendell-Rubin likelihood ratio test; LMR adjusted LRT, Lo-Mendell-Rubin adjusted likelihood ratio test; PB LRT, parametric bootstrapped likelihood ratio test. The four-class solution and its model fit indices are boldfaced*.

Supplementary Table 2 presents the sample characteristics of the fMRI subsample (*N* = 5,948; 52.9% female) and the respective LCA-derived groups based on irritability and ADHD symptoms. The mean age of the total sample was 9.9 years (SD = 0.6 years). Most youths were white (78.4%) and had caregivers attaining bachelor's degrees (29.9%). Most caregivers were married (71.7%) and had a total combined family income of $100,000–$199,999.

Comparisons of sex proportions across the latent classes suggested that there were more boys than girls in the two ADHD groups, [$\chi_{\left(3\right)}^{2}$ = 166.62, *p* < 0.001]. Specifically, relative to the typically developing group (45.4%), more boys were in the high ADHD symptoms with co-occurring irritability group (66.1%), [$\chi_{\left(1\right)}^{2}$ = 53.64, *p* < 0.001], and the moderate ADHD symptoms but low irritability group (56.0%), [$\chi_{\left(1\right)}^{2}$ = 19.14, *p* < 0.001]. The proportion of boys and girls in the high irritability but low ADHD group were comparable to typically developing peers, [$\chi_{\left(1\right)}^{2}$ = 0.09, *p* = 0.78], with the two groups having a slightly higher proportion of girls (i.e., 53.7 and 54.6%, respectively).

Additionally, there were more youths whose ethnicity was reported as not Hispanic or Latinx in the group with high ADHD symptoms with co-occurring irritability (83.9%) than in the typically developing group (78.7%), [$\chi_{\left(2\right)}^{2}$ = 21.37, *p* < 0.001]. There was also a significant group difference in caregiver education, in which most caregivers of youths with moderate ADHD symptoms but low irritability attained some college or associate degrees (34.6%), while most caregivers of typically developing youths attained bachelor's degrees (30.1%), [$\chi_{\left(5\right)}^{2}$ = 21.23, *p* < 0.001].

### Behavioral Performance

The mean SSRT was 262.29 ms (SD = 65.98 ms). Linear mixed model revealed no significant group differences in SSRT [*F*_(3,5246)_ = 0.52, *p* = 0.67].

### Response Inhibition and Error Processing Neural Coactivation Networks

#### Response Inhibition

**Figure 3:**
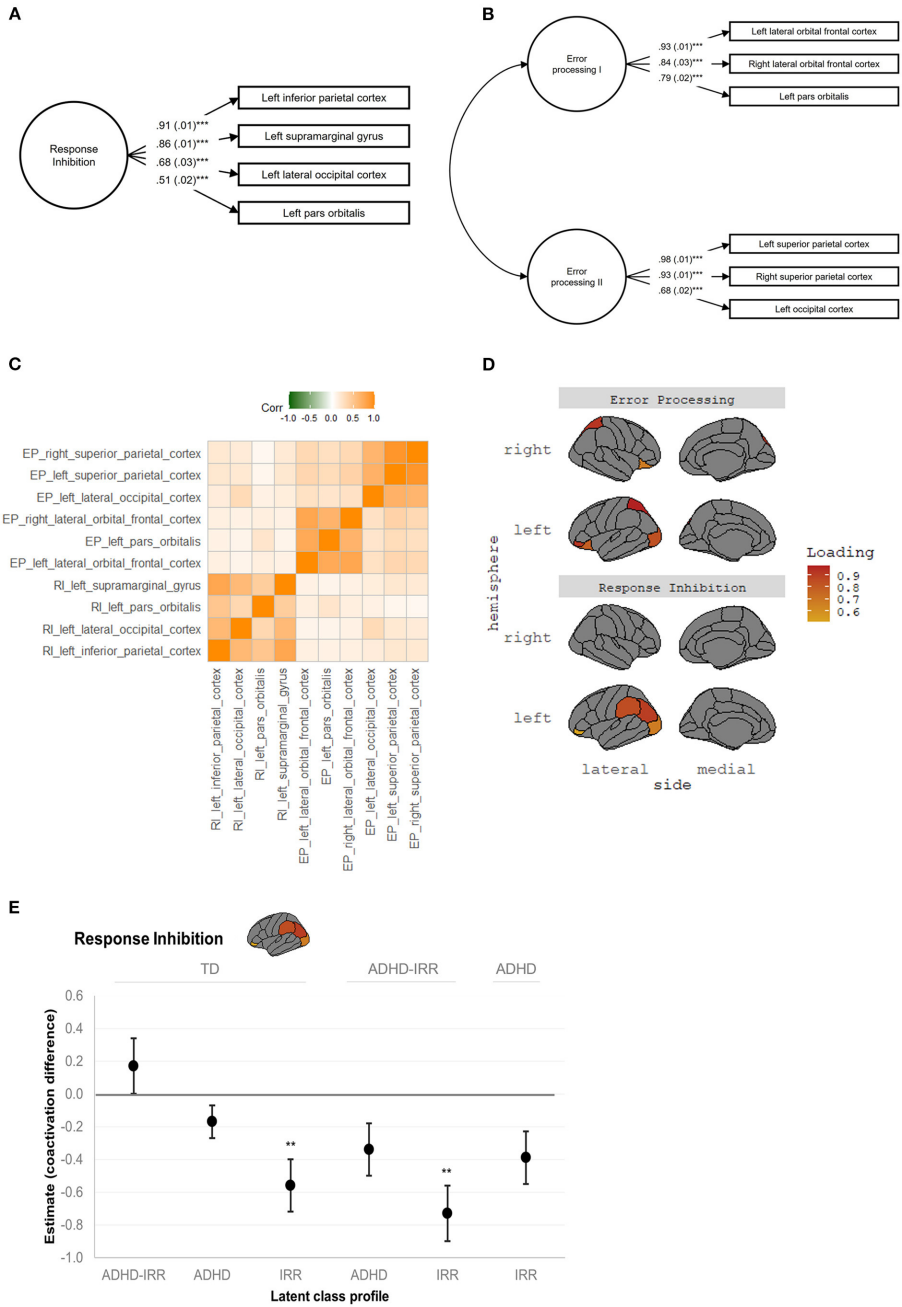
Neural coactivation networks of response inhibition and error processing. RI, response inhibition; EP, error processing. **(A)** Response inhibition coactivation network and constituent brain regions. **(B)** Error processing coactivation network and constituent brain regions. Standardized estimates and standard errors were shown in both **(A,B)**, all ***ps < 0.001. **(C)** Correlation matrix revealed uniqueness between the latent response inhibition factor and latent error processing factors I and II, and coherence within each latent factor. The correlation matrix was based on raw average beta weights of the constituent brain regions. **(D)** Visualization of the anatomical location and factor loadings of the response inhibition coactivation network and the error processing coactivation network. **(E)** Visualization of the significant group comparisons in the response inhibition network. Unstandardized estimates and standard errors are shown. Estimates represent the difference in neural coactivation during response inhibition relative to the reference group indicated on top. Positive estimates represent increased coactivation while negative estimates represent decreased coactivation relative to the reference group, respectively. ADHD, attention-deficit/hyperactivity disorder; IRR, irritability; TD, typically developing. **p ≤ 0.01, ***p < 0.001.

Exploratory factor analysis and subsequent confirmatory factor analysis confirmed a neural coactivation network consisting of a single latent response inhibition factor with excellent model fit, [$\chi_{\left(23\right)}^{2}$ = 59.62, *p* < 0.001, CFI = 0.99, TLI = 0.99, RMSEA = 0.02 [0.01, 0.02], SRMR = 0.01. The measurement models of the neural coactivation network of response inhibition achieved measurement invariance across the latent classes, all ΔCFI <0.01 and ΔRMSEA < 0.015 (Supplementary Table 4). Figure 3A depicts the factor structure of the response inhibition coactivation network and factor loadings of the respective brain regions. Briefly, the latent response inhibition factor included four regions, characterized by significant factor loadings (all *p*s < 0.001) in the left inferior parietal cortex (0.91), left supramarginal gyrus (0.86), left lateral occipital cortex (0.68), and left pars orbitalis (0.51).

#### Error Processing

Exploratory factor analysis and subsequent confirmatory factor analysis suggested a neural coactivation network consisting of two latent error processing factors with excellent model fit, [$\chi_{\left(36\right)}^{2}$ = 56.79, *p* < 0.001, CFI = 1.00, TLI = 0.99, RMSEA = 0.01 [0.01, 0.02], SRMR = 0.01. This error processing coactivation network also showed measurement invariance among the latent classes, all ΔCFI < 0.01 and ΔRMSEA < 0.015 (Supplementary Table 5). Figure 3B depicts the factor structure of the error processing coactivation network and factor loadings of the constituent brain regions. The first latent error processing factor was indicated by three regions, characterized by significant factor loadings (all *p*s < 0.001) in the bilateral lateral orbital frontal cortices (right = 0.84; left = 0.93) and left pars orbitalis (0.79). The second latent error processing factor was also indicated by three regions, characterized by significant factor loadings (all *p*s < 0.001) in the bilateral superior parietal cortices (right = 0.93; left = 0.98) and lateral occipital cortex (0.68).

Figure 3C presents the correlation matrix of the response inhibition coactivation network and the error processing network, which revealed the uniqueness of the latent response inhibition factor and the two latent error processing factors as well as the coherence within each latent factor. Figure 3D visualizes the anatomical location of the response inhibition coactivation network and the error processing coactivation network.

### Differential Group Differences in Neural Coactivation Patterns

#### Response Inhibition

Pairwise comparisons revealed significant group differences in activation of the response inhibition coactivation network (Table 2). Figure 3E visualizes the estimated *difference* in neural coactivation during response inhibition relative to the reference group in each pairwise comparison. Specifically, compared to typically developing peers (latent intercept set to 0), youths with high irritability but low ADHD symptoms (latent intercept = −0.56) showed decreased coactivation in the response inhibition network during the SST (difference estimate = −0.56, SE = 0.16, *p* = 0.01). Moreover, compared to youths with high ADHD symptoms with co-occurring irritability (latent intercept = 0.17), youths with high irritability but low ADHD symptoms (latent intercept = −0.56) showed further decreased coactivation during response inhibition (difference estimate = −0.73, SE = 0.17, *p* = 0.01). Finally, compared to youths with moderate ADHD but low irritability symptoms (latent intercept = −0.17), youths with high irritability but low ADHD symptoms (latent intercept = −0.56) showed decreased coactivation in the response inhibition network at a trend level (difference estimate = −0.39, SE = 0.16, *p* = 0.07).

**Table 2:** Comparison of latent brain activation patterns across groups.

	**Group 1 intercept**	**Group 2 intercept**	**Group 2–Group 1 estimate**	**SE**	***p***	***d***
**Response inhibition**						
TD vs. ADHD-IRR	0.00	0.17	0.17	0.17	0.77	0.41
TD vs. ADHD	0.00	−0.17	−0.17	0.10	0.31	0.54
TD vs. IRR	0.00	−0.56	−0.56	0.16	**0.01**	1.40
ADHD-IRR vs. ADHD	0.17	−0.17	−0.34	0.16	0.14	0.85
ADHD-IRR vs. IRR	0.17	−0.56	−0.73	0.17	**0.01**	1.77
ADHD vs. IRR	−0.17	−0.56	−0.39	0.16	0.07	0.98
**Error processing I**						
TD vs. ADHD-IRR	0.00	−0.13	−0.13	0.28	0.90	0.25
TD vs. ADHD	0.00	0.06	0.06	0.41	0.90	0.09
TD vs. IRR	0.00	0.20	0.20	0.54	0.90	0.27
ADHD-IRR vs. ADHD	−0.13	0.06	0.19	0.52	0.90	0.26
ADHD-IRR vs. IRR	−0.13	0.20	0.33	0.69	0.90	0.40
ADHD vs. IRR	0.06	0.20	0.14	0.60	0.90	0.18
**Error processing II**						
TD vs. ADHD-IRR	0.00	−0.06	−0.06	0.21	0.90	0.13
TD vs. ADHD	0.00	−0.21	−0.21	0.14	0.41	0.56
TD vs. IRR	0.00	−0.11	−0.11	0.28	0.90	0.21
ADHD-IRR vs. ADHD	−0.06	−0.21	−0.14	0.20	0.90	0.31
ADHD-IRR vs. IRR	−0.06	−0.11	−0.04	0.33	0.90	0.07
ADHD vs. IRR	−0.21	−0.11	0.10	0.28	0.90	0.19

*ADHD, attention-deficit/hyperactivity disorder; IRR, irritability; TD, typically developing. Results were adjusted for child's age, sex, race, ethnicity, caregiver's education, caregiver's marital status, total family income (past 12 months), and scan site. Unstandardized estimates were shown. Statistically significant comparisons surviving false discovery rate correction are boldfaced*.

#### Error Processing

No significant group differences were found in the error processing coactivation network (Table 2). Specifically, pairwise comparisons of error processing factor I, characterized by coactivation in the bilateral lateral orbital frontal cortices and pars orbitalis, found no significant group differences among the latent classes, estimates ranged from −0.13 to.33, *p*s = 0.90 after FDR-correction for multiple comparisons. Likewise, pairwise comparisons of error processing factor II, characterized by coactivation in the bilateral superior parietal cortices and occipital cortex, showed no significant group differences between the latent classes, estimates ranged from −0.21 to 0.10, *p*s ranged from 0.41 to 0.90.

### Group Differences in Regional Brain Activation

#### Response Inhibition

Linear mixed models revealed no significant group differences in activation of individual brain regions in the response inhibition network, *F*s ranged from 1.30 to 2.45, *p*s ranged from 0.18 to 0.34 (see Table 3).

**Table 3:** Group comparison of individual region of interest.

	**Sum of square**	**Mean square**	**Num df**	**Den df**	***F***	***p***	**η^2^**
**Response inhibition**							
L Inferior parietal cortex	0.07	0.02	3	5,265	1.42	0.34	0.78
L Lateral occipital cortex	0.26	0.09	3	5,261	2.09	0.20	0.74
L Pars orbitalis	0.31	0.10	3	5,263	2.45	0.18	0.76
L Supramarginal gyrus	0.06	0.02	3	5,264	1.30	0.34	0.75
**Error processing I**							
L Lateral orbital frontal cortex	0.75	0.25	3	5,265	2.41	0.18	0.75
L Pars orbitalis	2.76	0.92	3	5,263	4.75	**0.03**	0.75
R Lateral orbital frontal cortex	0.98	0.33	3	5,257	3.07	0.15	0.75
**Error processing II**							
L Lateral occipital cortex	0.30	0.10	3	5,264	1.93	0.20	0.75
L Superior parietal cortex	0.02	0.01	3	5,265	0.34	0.80	0.67
R Superior parietal cortex	0.06	0.02	3	5,264	0.86	0.51	0.75

*L, left hemisphere; R, right hemisphere. Results were adjusted for child's age, sex, race, ethnicity, caregiver's education, caregiver's marital status, total family income (past 12 months), and scan site. Statistically significant comparisons surviving false discovery rate correction are boldfaced*.

#### Error Processing

**Figure 4:**
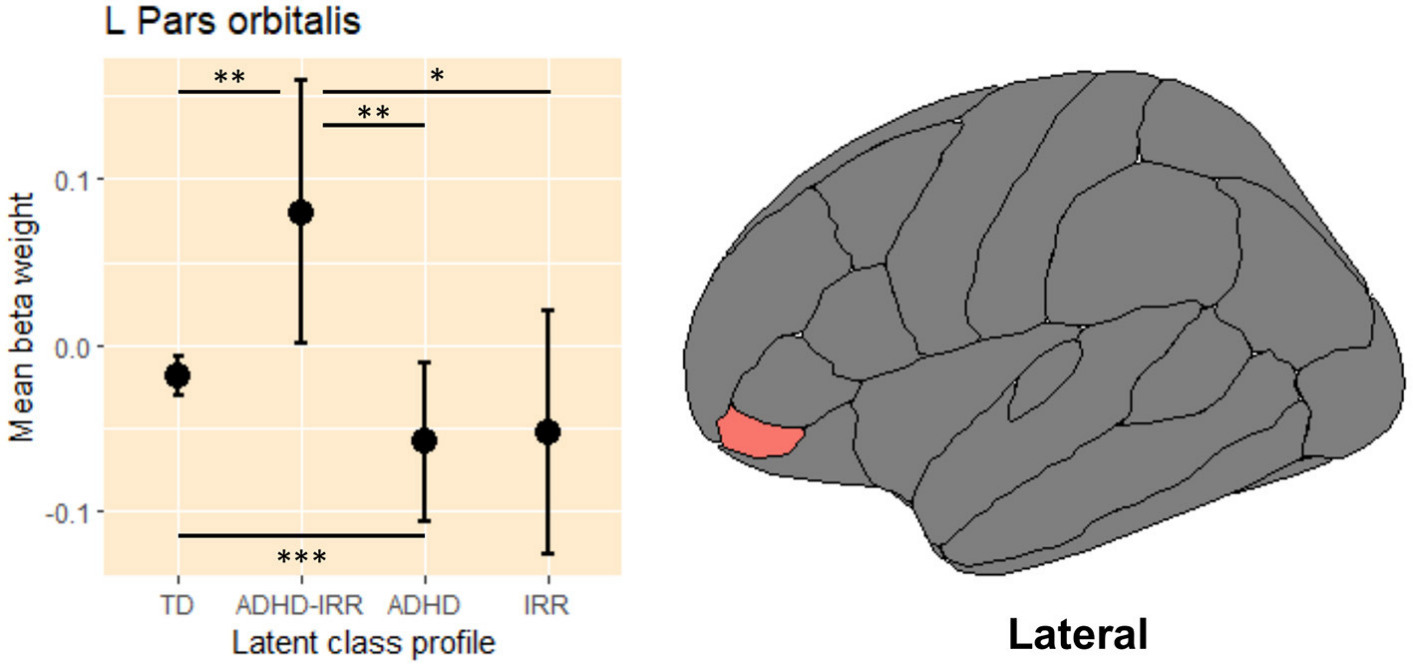
Average beta weights of regional activation in the left pars orbitalis during error processing. L, left hemisphere; ADHD, attention-deficit/hyperactivity disorder; IRR, irritability; TD, typically developing. Raw average beta weights and standard errors of regional activation in the left pars orbitalis during error processing across the four latent classes of youths. *p ≤ 0.05, **p ≤ 0.01, *** *p* < 0.001.

Linear mixed models revealed only one significant group difference in the left pars orbitalis activation, [*F*_(3,5263)_ = 4.75, *p* = 0.03; Table 3]. Figure 4 presents the raw means and standard errors of the average beta weights in this region. Pairwise comparisons indicated that relative to typically developing peers, youths with high ADHD symptoms with co-occurring irritability showed increased activation in the left pars orbitalis during error processing. Relative to youths with high ADHD with co-occurring irritability, youths with moderate ADHD but low irritability symptoms and youths with high irritability but low ADHD symptoms both showed decreased regional activation in the left pars orbitalis during error processing.

### Supplementary Results

#### Neural Correlates Across Latent Phenotypes Using All Available Brain Regions

Following the same modeling procedures but with the use of the full set of brain regions (i.e., 34 bilateral regions) during response inhibition and error processing on the SST (see Supplementary Tables 6, 7 for model fit and measurement invariance for response inhibition and error processing, respectively), group comparisons revealed some converging results as compared to the main results using selected regions with at least moderate reliability. While we observed a similar coactivation pattern of group differences during response inhibition as the main results above, the coactivation network comprised five different regions, namely the right pericalcarine cortex, bilateral lingual gyri and bilateral cuneus (Supplementary Figure 1A). Specifically, youths with high ADHD and co-occurring irritability showed increases in coactivation, while youths with moderate ADHD but low irritability symptoms and those with high irritability but low ADHD symptoms showed decreases in coactivation. However, these group differences were not statistically significant after FDR-corrected for multiple comparisons. The error processing network consisted of four regions (Supplementary Figure 1B), namely the bilateral superior frontal cortices, left caudal middle-frontal cortex, and right caudal anterior cingulate cortex, which partly overlapped with the salience network supporting executive control in response to environmental irregularities (68, 69). As in the main results analyzing a priori regions, no significant group differences emerged for error processing.

#### No Sex by Latent Phenotypes Interactions

Sex by group interactions on each of the constituent brain regions of the response inhibition and error processing coactivation networks suggested no significant sex-related group differences across all the individual brain regions, *F*s ranged from 0.20 to 2.77, *p*s ranged from 0.40 to 0.90 (Supplementary Table 9). See Supplementary Materials for detailed discussion.

#### Resembling Regional-of-Interest and Whole-Brain Results With Family Clustering

Significant results remained unchanged while accounting for family clustering (see Supplementary Tables 10–16 and Supplementary Figure 2).

#### *Post-hoc* Group Comparisons Removing All Sociodemographic Covariates

The latent group comparison on pre-selected brain regions showed no significant differences for response inhibition after FDR-correction; yet two neural differences emerged between the ADHD + irritability group and typically developing peers, and the ADHD only group for error processing I (Supplementary Table 17). Whole-brain group comparisons remained non-significant (Supplementary Table 18).

## Discussion

Using a population-based sample in the ABCD study at baseline, we applied data-driven, latent modeling techniques to identify shared and non-shared neural correlates of response inhibition and error processing in youths with distinct profiles of ADHD and irritability symptoms within a narrow developmental window (ages 9–10 years). This study is unique, since most prior neuroimaging studies in irritability included small sample sizes and therefore lacked power to compare across groups with distinct symptom profiles of ADHD and/or irritability and their neural correlates on the stop signal inhibition task. Also, the large ABCD dataset allowed for latent modeling of task-dependent neuroimaging measures. Such modeling was multivariate and estimated latent neural variables from observed neural responses, which reduces measurement errors, compared to conventional univariate analysis of neural responses (28). Using parent-reported ADHD and irritability symptoms, we identified four latent clinical phenotypes, namely youths with high ADHD with co-occurring irritability, moderate ADHD but low irritability symptoms, high irritability but low ADHD symptoms, and typically developing youths with low ADHD and low irritability symptoms. Main results suggested that the four LCA-derived groups differed in the response inhibition, but not error processing, coactivation network. These group differences were marked by decreased coactivation in youths with high irritability but low ADHD symptoms, relative to youths with high ADHD symptoms and co-occurring irritability and typically developing youths during response inhibition. This response inhibition network involved coactivation in the left inferior parietal cortex, left supramarginal gyrus, left lateral occipital cortex, and left pars orbitalis. We also observed primarily medium to large effect sizes for these analyses, suggesting that these neural differences were pronounced in this large study sample. Together, these results provide initial evidence of differential neural correlates during response inhibition across distinct phenotypic profiles of ADHD and irritability, paving the way for transdiagnostic interventions and precision medicine targeting neural mechanisms unique to specific symptom profiles.

### Distinct ADHD and Irritability Profiles

Based on the full baseline sample in the ABCD study, we found that 2.4% of youths had high levels of irritability symptoms but low ADHD symptoms. This estimate excluded youths with co-occurring ADHD symptoms and thus is slightly lower compared to the past epidemiological studies in school-age children with and without ADHD (70–72), but it is higher than the estimates in past studies where the full DMDD criteria were strictly applied (33, 73). In addition, we identified a group with comorbid high ADHD symptoms and co-occurring irritability, with a proportion of 6.7%. There was also a group of youths with moderate ADHD symptoms but low irritability (7.7%). Importantly, the proportion of the ADHD + irritability phenotype is significant because it indicates that almost half (46.6%) of the youths with ADHD symptoms in this large population-based study also have some degree of irritability—although at a lower level, but with greater variability, of irritability severity than youths with high irritability symptoms only. Indeed, it is estimated that up to 35%+ of youths in the community samples, and up to 70%+ in the clinical samples, have both severe chronic irritability/DMDD and ADHD (4, 41). Compared to youth with one condition alone, co-occurring ADHD and irritability are associated with greater symptom severity (9, 74), cause greater impairment, and exacerbate caregiver stress and mental health risks (5, 7, 9, 75, 76). Comorbidities may be particularly detrimental to young school-age children (e.g., the 9–10-year-olds in this sample), as interacting and forming relationships with peers and those outside the home environment are an important developmental task in this developmental stage. Symptoms of inattention, hyperactivity/impulsivity, and irritability or temper outbursts may make navigating the social world even more challenging for young children (77, 78).

### Sex Differences in Latent Phenotypes

Consistent with previous work (31), we found significant sex differences among the ADHD groups identified by the LCA. Compared to typically developing peers, boys were significantly more likely than girls to be classified in the two ADHD groups. These sex differences were consistent with the ADHD literature that there are sex differences observed in the reporting and clinical diagnosis of ADHD symptoms (79). One plausible factor related to these sex differences might be the current choice of parent-reported assessment as caregivers and teachers tend to rate ADHD symptoms as more impairing in boys than girls, and questions remain as to reporting/recognizing ADHD symptoms in girls (79). Yet, some suggested that the female genetics might be a protective factor against the development of ADHD symptoms (80). For irritability, we found that the sex distribution in the group with high irritability symptoms did not differ from that in the typically developing peers, largely consistent with past studies in community samples (34, 81). At a neural level, we also found no sex differences in regional activation during response inhibition and error processing across the latent phenotypes. However, we acknowledged that sex differences in neural responses may emerge during puberty (82), which requires further investigation at later time points in the ABCD study.

### Group Differences in Response Inhibition Coactivation Network

We found shared and non-shared neural correlates among youths with distinct profiles of ADHD and irritability symptoms. Specifically, the four LCA groups differed in the latent neural coactivation network for response inhibition, but not for error processing. Both response inhibition and error processing are relevant to the symptomatology of ADHD and irritability (3, 83). The current findings suggest that the neural mechanisms mediating inhibitory control over maladaptive behaviors might be different among the ADHD, irritability, and the comorbid phenotypes. The response inhibition coactivation network consists of the left inferior parietal cortex and left supramarginal gyrus, both of which support attentional control and sensorimotor integration (84) as well as the left lateral occipital cortex and left pars orbitalis (a subregion of the inferior frontal gyrus), which are part of the canonical inhibitory circuit important for attention control and orienting [e.g., (85, 86)].

Successful inhibition of one's behaviors largely depends on the accurate and efficient processing of sensorimotor signals, which allows for the planning and execution of inhibitory behavior (87, 88). Inefficient processing of early sensorimotor signals might contribute to poor and inefficient coordination of inhibitory behaviors (87), which corresponds to the inattentive symptoms and lack of motor inhibition present in ADHD (85, 89).

Relative to the large ADHD literature, functional neuroimaging research (via fNIRS and task-fMRI) on the role of response inhibition in irritability is small but growing. It has been suggested that the manifestation of temper outbursts and frustration can sometimes be a reactive response linked to failed inhibition of behaviors in response to changes in environmental demands (2, 3, 22). However, research on the neural circuitry that mediates ineffective inhibitory control in irritability remains limited. While some studies found altered regional activation in the frontal and parietal cortices important for top-down sensorimotor control and coordination (20, 21, 24), other studies reported disrupted salience-driven pathways in regions such as the anterior cingulate cortex and amygdala [e.g., (22)]. Both our main and supplementary results seem to provide more support to the former such that when comparing with the ADHD + irritability group and typically developing peers, youths with high irritability symptoms only showed consistently decreased coactivation in the frontal and parietal networks associated with attention control and sensorimotor coordination (84, 86). Using predictive modeling, a recent study (in youths with varying levels of transdiagnostic irritability, including youths with ADHD) demonstrated that the predictive networks of irritability primarily involved the sensorimotor networks and between those and the frontoparietal networks while performing a frustrating cognitive flexibility task (23), which is partly consistent with the current results from a systems neuroscience perspective.

The finding that there was hyper-coactivation in the ADHD + irritability group is novel, potentially indicative of effortful neural processing for attaining normative response inhibition. On the other hand, hypo-coactivation could reflect neural inefficiency for response inhibition in youths with high irritability symptoms alone, representing a differential neural profile. That aside, our findings raised further questions regarding the lack of neural differences between the ADHD groups and typically developing peers. Besides analytical differences and a narrower age window than past studies [e.g., (17)], the ADHD fMRI literature is heterogenous, and convergent activation patterns are yet to be established across neurocognitive domains in ADHD, including inhibitory control (19). Recent studies have also suggested that more refined experimental contrasts probing subordinate inhibitory functions are better suited to differentiate functional neural differences in ADHD from typically developing peers [e.g., sustained attention during response inhibition in 18 and sustained attention alone in (25)]. Nonetheless, it is noteworthy that our effect size estimates for these group comparisons were medium, warranting future replication. We also did not find any neural differences between the ADHD + irritability group and the ADHD only group, as compared to the one study that reported altered regional activations between the two ADHD groups during sustained attention (25). Yet, it is still early stage to conclude that these are contradicting findings since the current SST was not specifically designed to test sustained attention and sample size was small in (25), i.e., 31 DMDD with ADHD and 25 ADHD cases. We speculated that the neural differences between ADHD and ADHD + irritability are subtle and may be implicated in the interactive process between attention control and inhibitory control.

No behavioral differences (i.e., SSRT) were observed between the latent groups, suggesting that the current neural differences may not necessarily reflect response inhibition performance. However, it is premature to conclude that behavioral inhibitory deficits are not present in the ADHD and/or irritability phenotypes as inhibitory control was only indexed by SSRT. One should interpret these SSRT results with caution as it has been shown that the current SST violated the key assumption of context independence, which might compromise the interpretability of the integration estimates of SSRT. Alternative models accounting for context independence with less biased SSRT estimates are being developed [e.g., (90)].

### Error Processing Coactivation Network

Our main findings revealed no significant group differences in the error processing coactivation network across youths with distinct profiles of ADHD and irritability symptoms. The architecture of this error processing coactivation network consists of two smaller but interlinked networks. The first network was primarily within the frontal regions, including bilateral lateral orbital frontal cortices and left pars orbitalis. Although some suggested that these regions also constitute part of an attention network (91), these coactivation patterns during error processing likely reflect an alternative attention process that supports the detection of stimulus deviations relative to expected regularities (92), which is different from the attention process involved during response inhibition. Indeed, a study using a factorial go/no-go task found that response inhibition also involves sustained attention control (18), which is functionally distinct from error detection. Therefore, while prior research suggested there is neural inefficiency associated with inattentive symptoms in ADHD (10), the neural circuitry involved likely varies depending on the specific type of attention process required in a given experimental task or contrast. Interestingly, our supplementary analyses revealed hyper-coactivation in the first error processing network in the ADHD + irritability group relative to typically developing peers and the ADHD only group, although these *post-hoc* comparisons were not adjusted for any sociodemographic covariates and should therefore be interpreted with caution. The second constituent network of error processing was primarily within the parietal regions, represented by the bilateral superior parietal cortices and the left occipital cortex. The superior parietal cortices support manipulation of information stored in working memory (93), a critical aspect of executive functions. Along with coactivation in the left occipital cortex, a well-researched area critical for visual attention and processing (94), this second error processing coactivation network might be related to the computational work of comparing between environmental signals received and expected regularities in the environment during error processing.

Null findings aside, studying error processing and its associated neural coactivation can still be fruitful to elucidating the neural pathogenesis of ADHD and irritability. Of note, error processing was tested in a “cold”, non-emotional SST in this study, which has no affective or “hot” components that necessarily engage top-down emotion regulatory processes (95), although 50% error rate in the SST may evoke slight frustration [see (23)]. It is plausible that group differences might emerge in experimental tasks under an explicit emotional context that demands emotion regulation such as during a frustrative error processing task [see (23) for a modified Stop signal task]. Failing to detect violation of regularities in the surroundings can result in heightened emotional responses. Frustrative non-reward, a key neural dysfunction identified in youths with irritability (96), can be thought of as the processing of frustrative events that are erroneously against the expected reward schedule, which might involve error processing during frustration, warranting future research.

### Limited Group Differences in Regional Activation

To make results comparable to previous studies investigating regional activations, we examined group differences in each of the 10 constituent brain regions (FDR-corrected) that contributed to the response inhibition and error processing coactivation networks. Intriguingly, the conventional regional analysis revealed no significant group difference in each of the response inhibition regions, with one exception the left pars orbitalis (an anatomical subregion in the inferior frontal gyrus) during error processing. That is, relative to typically developing peers, youths with ADHD and occurring irritability showed heightened activation in the left pars orbitalis, whereas youths with ADHD only and with irritability only showed reduced activation in this region. The current lack of regional evidence might seem at odds with the previous studies that found an association between irritability severity and aberrant cognitive control-related neural responses in various frontal and subcortical regions (22, 24). However, this discrepancy may be due to differences in contrasts and tasks across studies—"incorrect stop vs. correct go” (error processing) during SST in the current study, as compared to “correct stop vs. correct go” (response inhibition) during SST in Chaarani et al. (24) and “congruent vs. incongruent trials” during the Flanker task in Liuzzi et al. (22). In addition, the current study was conducted in a late-childhood/pre-adolescent sample (i.e., 9–10-year-olds), whereas previous studies were in adolescents [i.e., 14-year-olds; (24)] and in a sample with a wide age range [i.e., 9–19 years; (22)].

Still, the functional significance of the pars orbitalis subregion of the inferior frontal gyrus has been proposed as a hub for attention control during error detection (97, 98) and shown to differentiate between youths with ADHD and peers with co-occurring conditions both in terms of functional activation [e.g., (86)] and volumetric differences [e.g., (99)]. Thus, at least at a regional level, the current finding suggests that the pars orbitalis and its involvement in error processing might be an important region of interest where activation differentiates between youths with varied profiles of ADHD and/or irritability, and typically developing youths.

### Clinical Implications

The latent classification findings and the shared and non-shared neural correlates of ADHD and/or irritability symptoms have important clinical implications. The current study identified a significant proportion of youths with ADHD and co-occurring irritability at baseline in the ABCD study, comprising almost half of the youths with at least moderate levels of ADHD symptoms. Despite the high prevalence of ADHD plus irritability and greater impairment, most interventions for youths with ADHD target ADHD symptoms and executive functions such as attention control and motor control, with less focuses on improving emotion regulation or mood symptoms (100), although stimulants seem to reduce, to some degree, irritability symptoms (101–103). However, it is noteworthy that inhibitory control and emotion regulation are interlinked processes as inhibiting or controlling one's behavior is a critical processing for emotion regulation. We therefore advocate for individualized treatment options that also target emotion regulation for youths with ADHD and co-occurring irritability. Given that co-occurring irritability in ADHD increases risks for later anxiety and depression (8, 104), treatments that target irritability symptoms may help improve the long-term outcomes of ADHD. Moreover, given that irritability symptoms may exacerbate caregiver stress and mental health risks such as parental depression, adjunct caregiver interventions improving parenting skills and stress management can be especially beneficial (7, 75).

Differential group differences in the neural coactivation networks provided a more nuanced understanding that the neural mechanisms vary subtly between ADHD, irritability, and their co-occurring conditions, and are more associated with response inhibition than error processing at the systems neuroscience level. These neural differences are of large effect sizes, which may be clinically meaningful. Given that ADHD symptoms (inattention, hyperactivity/impulsivity) and irritability (frustration and temper outbursts) are linked to dysfunction in inhibitory control, targeting response inhibition and its underlying circuitry may therefore be a promising intervention approach for youths with ADHD and co-occurring irritability symptoms. Effortful neural processing (manifested as hyper-activation) in the ADHD + irritability group may imply that developing adaptive inhibitory control strategies could be prioritized. Conversely, neural inefficiency (manifested as hypo-activation) in the irritability only group may suggest that consistent training that promotes one's efficiency to inhibit temper outbursts may be more beneficial to youths with primarily irritable symptoms. Taken together, this study identified distinct phenotypic profiles based on ADHD and irritability symptoms and found dissociable neural correlates during inhibitory control across these phenotypes. These provide novel insights into the potential neural mechanisms for change that are specific to unique symptom profiles, paving the way for transdiagnostic interventions and precision medicine (105, 106).

### Limitations and Future Directions

We note several limitations. First, the current study is cross-sectional, with data from a narrow development window (i.e., ages 9–10 years). It is likely that cognitive control-related brain functions undergo rapid development at later developmental stages (107), and these age-dependent changes might result in reorganization of neural coactivation patterns that are different from the ones reported here in childhood. Follow-up studies with longitudinal data in the ABCD study are useful to clarify if there are developmental or puberty-related changes in these cognitive control networks and the bi-directional influences between these neural differences and future ADHD/irritability symptoms over time using multi-wave structural equation modeling. Second, as acknowledged earlier, the latent variable modeling procedures were selected based on regions showing at least moderate test-retest reliability in the same SST reported by Korucuoglu et al. (44). Given that Korucuoglu and colleagues' study (44) was conducted in a small sample of young female adults and focused on regional activation, these regions might not necessarily represent the most reliable regions in the current late childhood sample. Nonetheless, we presented supplementary modeling results based on all available brain regions in the dataset and found similar coactivation patterns in the ADHD and irritability groups compared to the typically-developing and irritability groups, although these group comparisons did not reach statistical significance after correcting for multiple comparisons (see Supplementary Materials). Third, the current study analyzed pre-processed activation beta weights in the ABCD dataset; future studies applying alternative anatomical parcels to the raw data may help validate the current neural coactivation networks with more refined brain regions specified. Moreover, future studies could employ nested cross-validation and machine learning approaches that split the ABCD sample into a train/validation set and a test set to validate the current findings and improve the generalizability of the findings. Future translational studies may link the current neural differences to prospective ADHD/irritability treatment outcomes in other clinical datasets. Fourth, as with other fMRI studies (45), our study is also potentially limited by the poor reliability of the fMRI measures, although we tried to mitigate this problem by pre-selecting regions with moderate to good test-retest reliability and using a multivariate modeling approach to derive neural coactivation networks. Fifth, it should be noted that the neural coactivation networks identified were not the equivalent of functional connectivity measures that are more conventionally used in the literature. On one hand, as compared to seed-based measures of functional connectivity, these neural coactivation networks were robustly derived on a data-driven basis, with reduced measurement errors, and allowed for group comparisons while considering the multivariate fMRI data structure [e.g., (27)]. On the other hand, whole-brain, non-seed based functional connectivity as well as structural connectivity using fractional anisotropy in diffusion tensor imaging may provide a more direct proxy of connectivity. However, diffusion tensor imaging may be limited in identifying neural alterations that are task-dependent, and architectural issues such as crossing and “kissing” fibers can compromise results interpretation (108). Sixth, the latent phenotypes were derived from parent-reported ADHD and irritability symptoms. Other symptom informants, such as teachers, might provide a more comprehensive presentation of the clinical phenotypes across settings. Finally, the SST used in the ABCD study was not designed to elicit any irritability-relevant states (e.g., frustration). A recent study found that irritability-related neural dysconnectivity are prominent when children are in a state of frustration (23). Future studies with “hot” executive function tasks, e.g., adding affective components to the standard SST design, in addition to “cold” executive functions, may help uncover neurocognitive markers that further differentiate between the ADHD, irritability, and the ADHD + irritability phenotypes.

## Conclusion

This study is the first to use the ABCD dataset to examine shared and non-shared neural correlates of response inhibition and error processing across distinct phenotypes of ADHD, irritability, and their co-occurrence using data-driven, latent variable modeling techniques. This is the largest study to date on irritability and neuroimaging. Taking a systems neuroscience and multivariate approach, differential group differences emerged in the neural coactivation network associated with response inhibition but not error processing, providing novel evidence for differential neural mechanisms among these common and frequently co-occurring clinical phenotypes. Findings may inform a precision medicine approach in which novel treatments for ADHD, irritability, and their comorbidity can be developed and tailored to each individual patient based on the extent to which the treatment targets a specific form of neural dysfunction.

## Data Availability Statement

All data analyzed in this study are accessible through an official request to the NIH Data Archive (https://nda.nih.gov/abcd). An NDA study detailing related information for this study may also be accessed at the following https://doi.org/10.15154/1524665 further inquiries can be directed to the corresponding author/s.

## Ethics Statement

The studies involving human participants were reviewed and approved by the Adolescent Brain Cognitive Development Study Consortium, National Institute of Health. Written informed consent to participate in this study was provided by the participants' legal guardian/next of kin.

## Author Contributions

KSL developed the research questions, analyzed the data, interpreted the results, and drafted the original manuscript. JL and JX curated the data under the National Institute of Mental Health Data Archive study ID: 9487, and reviewed the results and manuscript. EL and ZL reviewed the manuscript, provided critical comments on the intellectual content of the manuscript and overall supervision. W-LT developed the research questions, reviewed and provided critical comments on the data analysis and intellectual content of the manuscript, interpreted the results, and provided primary supervision. All authors agreed to be accountable for the content of the work. All authors contributed to the article and approved the submitted version.

## Funding

KL was supported by a doctoral student scholarship awarded by the Hong Kong Jockey Club Charities Trust. EL was supported by the National Institute of Mental Health Intramural Research Program (ZIAMH002781). W-LT was supported by a research grant from the National Institute of Mental Health (R00MH110570) and the Fund to Retain Clinical Scientists from the Yale School of Medicine and the Yale Center for Clinical Investigation. The ABCD Study® is supported by the National Institutes of Health and Additional Federal Partners under award numbers U01DA041048, U01DA050989, U01DA051016, U01DA041022, U01DA051018, U01DA051037, U01DA050987, U01DA041174, U01DA041106, U01DA041117, U01DA041028, U01DA041134, U01DA050988, U01DA051039, U01DA041156, U01DA041025, U01DA041120, U01DA051038, U01DA041148, U01DA041093, U01DA041089, U24DA041123, U24DA041147. A full list of supporters is available at https://abcdstudy.org/federal-partners.html. A listing of participating sites and a complete listing of the study investigators can be found at https://abcdstudy.org/scientists/workgroups/. The above stated funders play no role in the design, implementation, and writing of the study.

## Author Disclaimer

This study reflects the views of the authors and may not reflect the opinion or views of the funders and NIH or the ABCD consortium investigators.

## Conflict of Interest

The authors declare that the research was conducted in the absence of any commercial or financial relationships that could be construed as a potential conflict of interest. The reviewer PV-R declared a shared affiliation, with no collaboration, with the authors EL at the time of the review.

## Publisher's Note

All claims expressed in this article are solely those of the authors and do not necessarily represent those of their affiliated organizations, or those of the publisher, the editors and the reviewers. Any product that may be evaluated in this article, or claim that may be made by its manufacturer, is not guaranteed or endorsed by the publisher.
