# Supplementary material for: Characterizing the Neural Correlates of Response Inhibition and Error Processing in Children With Symptoms of Irritability and/or Attention-Deficit/Hyperactivity Disorder in the ABCD Study®
Source: Front Psychiatry. 2022 Mar 4;13:803891. doi: 10.3389/fpsyt.2022.803891 (PMC8931695; doi:10.3389/fpsyt.2022.803891)
Supplement: Supplementary file 1 [file Data_Sheet_1.docx]

Supplementary Materials

# Supplementary Data

**Sample selection and comparison of sample characteristics**

To maximize the sample size for classifying the latent class membership of youths based on their parent-reported ADHD and irritability symptoms, the latent class analysis (LCA) was first conducted in the full baseline sample on the K-SADS in the 3.0 Release (*N* = 11,875). Of the full sample, 127 cases lacked the sufficient information on the ADHD and irritability symptoms, which resulted in a corrected sample of 11,748 in the latent class analysis with occasionally missing information handled by full information maximum likelihood robust estimation. This strategy was to ensure that the latent class analysis would be most sufficiently powered, and that the classification of symptom profiles would be based on the entire spectrum of ADHD symptom and child irritability available across all participating youths, thereby bolstering generalizability of the classification results. Following the LCA, youths with complete and quality neuroimaging data, i.e., those who completed the Stop Signal Task (SST) scan with functional neuroimaging data surpassing quality control, were included in the subsequent analyses, including the latent variable modeling and multilevel modeling procedures. Consistent with prior work using the ABCD datasets (1-3), the current sample was selected based on various quality control parameters, motion effects, and screening of outliers in the functional neuroimaging data on the SST. As summarized in the main text, the quality control parameters were FreeSurfer quality control parameter (fsqc_qc), degrees of freedom across runs (tfmri_sa_beta_dof > 200), and average framewise displacement on the SST (tfmri_sa_beta_mm < 0.9mm). Based on (4-5) and after consultation with the ABCD Data Analysis and Informatics Center, various behavioral quality control parameters were also introduced to the screening, namely poor SST task performance (tfmri_sst_beh_performflag), task coding error indicated by the glitch flag (tfmri_sst_beh_glitchflag), and number of Stop trials with 0ms Stop Signal Delay (tfmri_sst_beh_0ssdcount < 10% of all Stop trials). The application of the various screening criteria was to balance between the generalizability of the current findings and the inclusion of quality data, as data of poor imaging or behavioral quality may introduce biases and noises and hurt the power of analyses. Missingness of these variables and socio-demographic data was handled by maximum likelihood estimation in Mplus.

The above screening resulted in a current sample of 5,948 youths. As commonly reported in previous work (e.g., 1-3), visual inspection and statistical group comparisons with the excluded sample (*N* = 5,930) revealed significant differences in all socio-demographic variables; and the same sociodemographic differences were observed among the included LCA groups, except for age (see Table S1 for a summary of the sample characteristics comparison and Table S2 for the sample characteristics of the included sample). All the socio-demographic variables were therefore included as covariates in the data analyses. Modeling results remained largely unchanged when removing age as a covariate, except for the group comparison using selected regions from (6) between typically developing peers and youths with high irritability symptoms only, which was significant at a trend level corrected for false discovery rate (FDR) (Estimate = .40, SE = .16, *p* = .09). The hierarchical structure introduced by scan sites was taken into account in the latent modeling procedures as described in the main text. Where applicable, the statistical procedures were corrected for multiple comparisons using FDR and significant at alpha = .05.

**Group comparison using all available brain regions**

Table S3 summarizes the full list of brain regions included in the main analysis and their respective variable names in the ABCD dataset, which were selected based on ICC > .60 from Korucuoglu and colleagues (6) (see Tables S4 and S5 for model fit and measurement invariance). While the work of Korucuoglu and colleagues (6) provided the much-needed evidence for the most robust brain regions that showed moderate to high test-retest reliability for response inhibition and error processing (termed error monitoring in the article) on the SST, we acknowledged that such evidence was based on a young adult sample, and hence might not be entirely applicable to younger children, such as those included in the current study. To supplement the main results based on selected regions from Korucuoglu and colleagues (6), the same series of model identification and latent modeling procedures were conducted with the use of all 34 bilateral sets of available brain regions in the SST dataset (7).

To derive coactivation network for response inhibition, exploratory factor analysis was conducted to identify indicators and factor structures. Subsequent confirmatory factor analysis revealed excellent model fit, χ^2^(29) = 49.27, *p* = .01, CFI = 1.00, TFI = 1.00, RMSEA = .01 [.01, .02], SRMR = .01, with measurement invariance achieved, ΔCFI> .01 and ΔRMSEA>.015 (8). Table S6 presents the model fit information and measurement invariance test results of the response inhibition coactivation network. This response inhibition coactivation network comprised of five regions (see Figure S1A), characterized by significant factor loadings (all *p*s <.001) in the right pericalcarine cortex (.93), bilateral lingual gyri (right = .92, left = .91), and bilateral cuneus (right = .85, left = .90).

Similarly, to derive coactivation network for error processing, confirmatory factor analysis, based on indicators and factor structures derived from exploratory factor analysis, suggested excellent model fit, χ^2^(22) = 114.24, *p* = <.001, CFI = .99, TFI = .99, RMSEA = .03 [.02, .03], SRMR = .01, with measurement invariance achieved, ΔCFI> .01 and ΔRMSEA>.015 (8). Table S7 presented the model fit information and measurement invariance test results of the error processing coactivation network. This error processing coactivation network was indicated by four regions (see Figure S1B) with significant factor loadings (all *p*s <.001), represented by the bilateral superior frontal cortices (right = .97, left = .91), left caudal middle frontal cortex (.84), and the right caudal anterior cingulate cortex (.72).

Table S8 summarizes the group comparison results of the latent neural coactivation networks across latent classes. The group comparison results were conducted using the model constraint and difference test procedures in Mplus version 8.3 (9), with all the socio-demographics and the hierarchical structure of scan sites adjusted for. While the same coactivation patterns were observed for response inhibition, where there was increased coactivation in youths with high ADHD symptoms and co-occurring irritability and decreased coactivation in youths with moderate ADHD symptoms only and in youths with high irritability symptoms only, these group differences did not reach statistical significance after FDR-corrected for multiple comparisons (Estimates ranged from -.54 to .31, *p*s = .12 to .66). As for error processing, the same null results were found across all developmental groups (Estimates ranged from -.44 to .16, *p*s ranged from .16 to .61). Results remained unchanged when excluding race and caregiver marital status as covariates (Estimates ranged from -.51 to .25, *p*s ranged from .21 to .89 for response inhibition; Estimates ranged from -.38 to .13, *p*s ranged from .13 to .21 for error processing).

**Sex by group interaction in regional brain activation**

The large sample in the ABCD study provided a unique opportunity to explore potential sex differences in the shared and nonshared neural correlates in youths affected by ADHD or irritability alone, and their co-occurrence. Research shows that boys with ADHD tend to be more hyperactive and have more difficulties in motor response inhibition and cognitive flexibility compared to girls with ADHD (for a meta-analysis, see 30); yet sex differences in irritability, with or without ADHD, and their neural correlates remain largely unknown due to limited and underpowered studies. Here, we tested if sex moderates the differences in each of the constituent brain regions of the response inhibition and error processing coactivation networks across the four latent classes. Because of the Mplus limitation that latent group comparisons are not available for multiple hierarchical structure yet, the sex by group interaction analysis was conducted on a regional level, which would nonetheless allow for some inspection of potential sex differences in the neural correlates across the latent phenotypes of ADHD and irritability. Following similar procedures as detailed in the main text, average beta weights of each of the selected brain regions (6) were fitted to a series of linear mixed models, with the sex by group interaction term introduced to the models. The models were adjusted for all the socio-demographic variables and scan site.

Table S9 summarized results of the sex by group interaction analysis. Results suggested that none of the constituent brain regions showed significant sex by group differences in activation level across latent classes for both response inhibition and error processing, *F*s ranged from .20 to 2.77, *p*s ranged from .40 to .90.

**Group comparison with family clustering**

We conducted post-hoc group comparisons of the latent neural coactivation network for response inhibition and error processing with family clustering. Specifically, the same modeling procedures were used as described in the main text (see **Methods**), except that the nesting structure of families was taken into account by using family ID as a clustering variable in Mplus, instead of scan site as presented in the main text. We first presented results using the a priori brain regions specified in Korucuoglu and colleagues (6), and then whole-brain results using all available brain regions in the SST dataset. Statistical significance of the multiple group comparisons was corrected using false discovery rate.

For the set of a priori selected brains, confirmatory factor analysis revealed excellent model fit, χ^2^(23) = 66.81, *p* < .001, CFI = .99, TFI = .99, RMSEA = .02 [.01, .02], SRMR = .01, with measurement invariance achieved, ΔCFI> .01 and ΔRMSEA>.015 (8) for response inhibition (Table S10).Excellent model fit, χ^2^(36) = 55.80, *p* = .02, CFI = 1.00, TFI = .99, RMSEA = .01 [.004, .02], SRMR = .01, and measurement invariance, ΔCFI> .01 and ΔRMSEA>.015 (8), were also observed for error processing (Table S11). Similar to the main results with scan site clustering, subsequent comparison of the latent brain coactivation patterns with the a priori regions revealed significant group differences for response inhibition. These group differences were marked by a significant decreased in neural coactivation in youths with irritability symptoms only relative to typically developing peers (Estimate = -.56, SE = .17, *p* = .01), and youths with high ADHD symptoms and co-occurring irritability (Estimate = -.73, SE = .23, *p* = .01). No significant group differences were observed for error processing, Estimates ranged from -.21 to .33, *p*s ranged from .49 to .90. Table S12 summarizes the estimates and effect sizes for these latent group comparisons. Further investigation of regional activation revealed that there was a significant group difference in the left pars orbitalis during error processing using linear mixed model with the random intercept for family nesting, *F*(3, 5260) = 4.80, *p* = .02 (Table S13). Specifically, there was a significant increased activation in the left pars orbitalis in youths with high ADHD symptoms and co-occurring irritability, relative to all other groups (Figure S2). No significant group differences were found for response inhibition (*F*s ranged from 1.14 to 2.31, *p*s ranged from .18 to .41), and the remaining indicating regions for error processing (*F*s ranged from .36 to 3.21, *p*s ranged from .67 to .75). See Table S13 for the regional estimates and effect sizes.

For the full set of whole-brain regions, both the neural coactivation networks for response inhibition, χ^2^(29) = 45.42, *p* = .03, CFI = 1.00, TFI = 1.00, RMSEA = .01 [.004, .02], SRMR = .01 (Table S14) and error processing, χ^2^(22) = 116.05, *p* < .001, CFI = .99, TFI = .99, RMSEA = .03 [.02, .03], SRMR = .01 (Table S15) revealed excellent model fit and achieved measurement invariance, ΔCFI> .01 and ΔRMSEA>.015 (8). Subsequent comparison of the latent brain coactivation patterns, however, revealed no significant group differences for response inhibition (Estimates ranged from -.54 to .31, *p*s ranged from .13 to .73), and error processing (Estimates ranged from -.44 to .16, *p*s ranged from .13 to .66). See Table S16 for the respective estimates and effect sizes of the whole-brain comparisons.

**Group comparison removing all covariates**

Given that various sociodemographic differences between the LCA groups were of small to medium effect sizes, we re-ran the main latent group comparisons on a post-hoc basis with all sociodemographic covariates removed from the modeling procedures. These comparisons were conducted for the pre-selected brain regions and across the whole brain on the SST.

Among the pre-selected brain regions (Table S17), the neural coactivation differences for response inhibition did not survive FDR-correction for multiple comparisons (Estimates ranged from -.001 to .02, *p*s ranged from .18 to .97). Interestingly, we found two significance group differences for the first latent coactivation factor of error processing, marked by hyper-activation in the ADHD + irritability group relative to typically developing peers (Estimate = .05, SE = .02, *p* = .03), and relative to the ADHD only group (Estimate = -.07, SE = .02, *p* = .02). Whole-brain results remained non-significant for response inhibition and error processing (Estimates ranged from -.02 to .01, *p*s ranged from .24 to 1.00, see Table S18).

# References

| 1 | Chaarani B, Hahn S, Allgaier N, Adise S, Owens MM, Juliano AC, et al. Baseline brain function in the preadolescents of the ABCD Study. *Nat Neurosci.* 2021;24(8):1176–86. doi:10.1038/s41593-021-00867-9 |
| --- | --- |
| 2 | Hawes SW, Waller R, Byrd AL, Bjork JM, Dick AS, Sutherland MT, et al. Reward processing in children with disruptive behavior disorders and callous-unemotional traits in the ABCD study. *Am J Psychiatry.* 2021;178(4):333–42. doi:10.1176/appi.ajp.2020.19101092 |
| 3 | Lichenstein SD, Roos C, Kohler R, Kiluk B, Carroll KM, Worhunsky PD, et al. Identification and validation of distinct latent neurodevelopmental profiles in the Adolescent Brain and Cognitive Development study. *Biol Psychiatry Cogn Neurosci Neuroimaging* [Internet]. 2021; Available from: http://dx.doi.org/10.1016/j.bpsc.2021.02.013 |
| 4 | Bissett PG, Hagen MP, Jones HM, Poldrack RA. Design issues and solutions for stop-signal data from the Adolescent Brain Cognitive Development (ABCD) study. *Elife* [Internet]. 2021;10. Available from: http://dx.doi.org/10.7554/eLife.60185 |
| 5 | Garavan H, Hahn S, Chaarani B, Juliano A, Allgaier N, Yuan DK, et al. The ABCD Stop Signal Data: Response to Bissett et al [Internet]. *bioRxiv.* 2020. Available from: http://dx.doi.org/10.1101/2020.07.27.223057 |
| 6 | Korucuoglu O, Harms MP, Astafiev SV, Golosheykin S, Kennedy JT, Barch DM, et al. Test-retest reliability of neural correlates of response inhibition and error monitoring: An fMRI study of a stop-signal task. *Front Neurosci.* 2021;15:624911. doi:10.3389/fnins.2021.624911 |
| 7 | Hagler DJ Jr, Hatton S, Cornejo MD, Makowski C, Fair DA, Dick AS, et al. Image processing and analysis methods for the Adolescent Brain Cognitive Development Study. *Neuroimage.* 2019;202(116091):116091. doi:10.1016/j.neuroimage.2019.116091 |
| 8 | Chen FF. Sensitivity of goodness of fit indexes to lack of measurement invariance. *Struct Equ Modeling.* 2007;14(3):464–504. doi:10.1080/10705510701301834 |
| 9 | Muthén L, Muthén B. Mplus (Version 8) [computer software]. 1998-2017. |

# Supplementary Figures and Tables

Because of limited space, the supplementary tables were uploaded separately, available online.

## Supplementary Figures

**Figure S1.** Neural coactivation networks of response inhibition and error processing based on all available brain regions


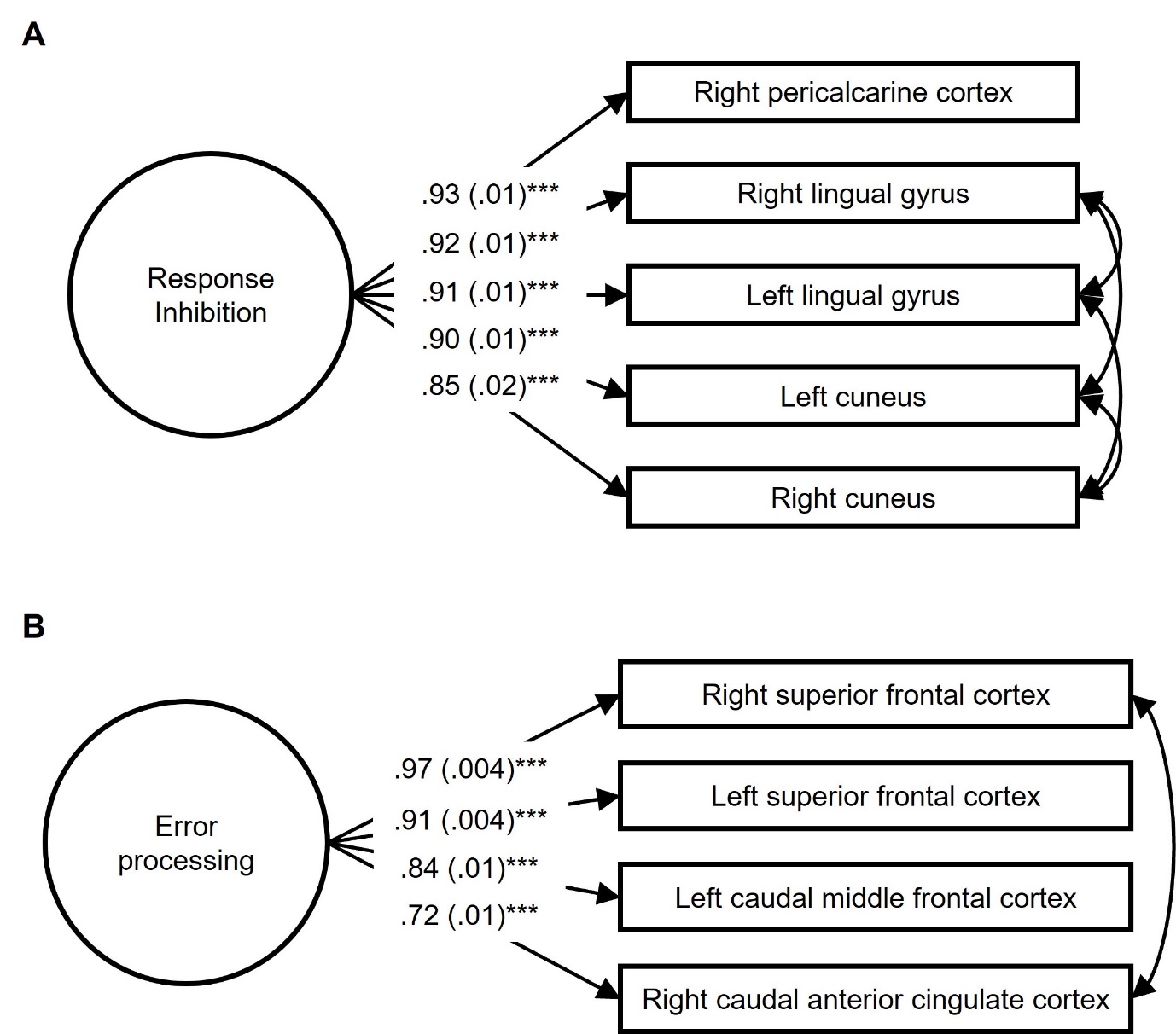


*Notes.* (A) Response inhibition coactivation network and constituent brain regions. (B) Error processing coactivation network and constituent brain regions. Standardized estimates and standard errors were showed, all ****p*s <.001. Paths covarying between individual brain regions within each of the coactivation networks were suggested by modification indices in Mplus.

**Figure S2.** Average beta weights of regional activation in the left pars orbitalis during error processing from family clustering analysis
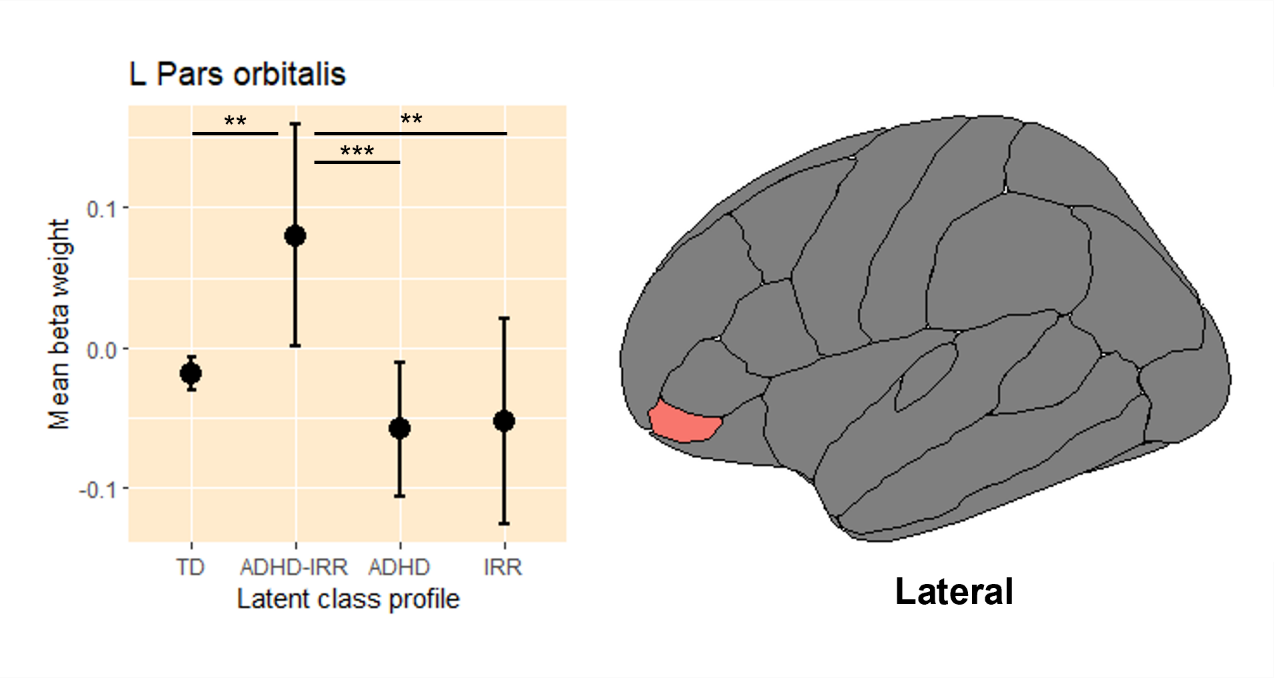


*Notes.* L = left hemisphere; ADHD = attention-deficit/hyperactivity disorder; IRR = irritability; TD = typically developing. Raw average beta weights and standard errors of regional activation in the left pars orbitalis during error processing across the four latent classes of youths. Results remained largely unchanged as compared to the main analysis with scan site clustering.

** *p* ≤.01, *** *p* <.001.
