# Supplementary material for: Characterizing the Neural Correlates of Response Inhibition and Error Processing in Children With Symptoms of Irritability and/or Attention-Deficit/Hyperactivity Disorder in the ABCD Study®
Source: Front Psychiatry. 2022 Mar 4;13:803891. doi: 10.3389/fpsyt.2022.803891 (PMC8931695; doi:10.3389/fpsyt.2022.803891)
Supplement: Supplementary file 2 [file Data_Sheet_2.pdf]

Table S1. Sample characteristics of included and excluded study participants

Variable	Total (Included) N=5,948 n	%	Excluded N=5,930 n	%	Comparison
Age (mean, SD in months)	119.3 (7.6)	--	118.6 (7.4)	--	t(11876)=-5.38, p<.001, d=.09
Age (mean, SD in years)	9.9 (0.6)	--	9.9 (0.6)	--	
Sex					χ2(1)=242.82, p<.001, V=.14
Female	3144	52.9	2538	42.8	
Male	2804	47.1	3392	57.2	
Race					χ2(6)=298.26, p<.001, V=.16
White	4665	78.4	4140	69.8	
Black/African American	791	13.3	1200	20.2	
American Indian/Alaska Indian	30	0.5	39	0.7	
Native Hawaiian/Guamanian/Samoan/Other Pacific Islander	13	0.2	11	0.2	
Asian	143	2.4	150	2.5	
Other race	233	3.9	292	4.9	
Refuse to answer/Don't know	73	1.2	98	1.7	
Ethnicity					χ2(2)=14.71, p<.001, V=.04
Hispanic/Latinx	1173	19.7	1238	20.9	
Not Hispanic/Latinx	4707	79.1	4607	77.7	
Refuse to answer/Don't know	68	1.1	85	1.4	
Caregiver education					χ2(5)=255.32, p<.001, V=.15
< High school diploma	204	3.4	280	4.7	
High school diploma/GED	551	9.3	709	12.0	
Some college/Associate degrees	1623	27.3	1865	31.5	
Bachelors	1781	29.9	1552	26.2	
Postgraduate degrees	1667	28.0	1327	22.4	
Refuse to answer/Don't know	122	2.1	197	3.3	
Caregiver marital status					χ2(6)=282.72, p<.001, V=.15
Married	4264	71.7	3727	62.8	
Widowed	45	0.8	52	0.9	
Divorced	513	8.6	569	9.6	
Separated	217	3.6	247	4.2	
Never married	582	9.8	878	14.8	
Living with partner	289	4.9	399	6.7	
Refuse to answer/Don't know	38	0.6	58	1.0	
Total combined family income (USD)					χ2(10)=357.88, p<.001, V=.17
Less than $5,000	148	2.5	269	4.5	
$5,000 through $11,999	166	2.8	255	4.3	
$12,000 through $15,999	127	2.1	147	2.5	
$16,000 through $24,999	211	3.5	313	5.3	
$25,000 through $34,999	312	5.2	342	5.8	
$35,000 through $49,999	439	7.4	495	8.3	
$50,000 through $74,999	723	12.2	776	13.1	
$75,000 through $99,999	840	14.1	732	12.3	
$100,000 through $199,999	1811	30.4	1504	25.4	
$200,000 and greater	719	12.1	531	9.0	
Refuse to answer/Don't know	452	7.6	566	9.5	

Notes. GED = General Equivalent Diploma.

Table S2. Sample characteristics of study participants

Variable	Typically-developing N=5,110			ADHD, Moderately irritable N=298			Moderate ADHD, Non-irritable N=393				Irritable, Non-ADHD N=147					Total (Included) N=5,948	
	n	%	Omnibus comparison	n	%	Comparison vs. TD	n	%	Comparison vs. TD	Comparison vs. ADHD-IRR	n	%	Comparison vs. TD	Comparison vs. ADHD-IRR	Comparison vs. ADHD	n	%
Age (mean, SD in months)	119.4 (7.5)	--	F(3, 5944)=2.95, p=.03, η²=.001	118.4 (7.8)	--	--	119.8 (7.4)	--	--		118.4 (7.9)	--	--			119.3 (7.6)	--
Age (mean, SD in years)	10.0 (0.6)	--	--	9.9 (0.7)	--	--	10.0 (0.6)	--	--		9.9 (0.7)	--	--			9.9 (0.6)	--
Sex			χ2(3)=166.62, p<.001, V=.14			χ2(1)=53.64, p<.001, V=.10			χ2(1)=19.14, p<.001, V=.06	χ2(1)=17.99, p<.001, V=.16			χ2(1)=.09, p=.78, V=.004	χ2(1)=25.85, p<.001, V=.24	χ2(1)=5.64, p=.07, V=.10		
Female	2791	54.6		101	33.9		173	44.0			79	53.7				3144	52.9
Male	2319	45.4		197	66.1		220	56.0			68	46.3				2804	47.1
Race			--			χ2(6)=6.00, p=.56, V=.03			χ2(6)=6.02, p=.56, V=.03	χ2(6)=7.32, p=.42, V=.10			χ2(6)=7.96, p=.38, V=.04	χ2(6)=9.70, p=.27, V=.15	χ2(6)=9.31, p=.27, V=.13		
White	3995	78.2		239	80.2		304	77.4			127	86.4				4665	78.4
Black/African American	673	13.2		41	13.8		63	16.0			14	9.5				791	13.3
American Indian/Alaska Indian	24	0.5		3	1.0		2	0.5			1	0.7				30	0.5
Native Hawaiian/Guamanian/Samoan/Other Pacific Islander	12	0.2		0	0.0		1	0.3			0	0.0				13	0.2
Asian	132	2.6		5	1.7		5	1.3			1	0.7				143	2.4
Other race	208	4.1		8	2.7		15	3.8			2	1.4				233	3.9
Refuse to answer/Don't know	66	1.3		2	0.7		3	0.8			2	1.4				73	1.2
Ethnicity			--			χ2(2)=21.37, p<.001, V=.06			χ2(2)=2.69, p=.42, V=.02	χ2(2)=16.01, p<.001, V=.15			χ2(2)=5.09, p=.19, V=.03	χ2(2)=1.40, p=.60, V=.06	χ2(2)=4.42, p=.23, V=.09		
Hispanic/Latinx	1038	20.3		39	13.1		77	19.6			19	12.9				1173	19.7
Not Hispanic/Latinx	4022	78.7		250	83.9		309	78.6			126	85.7				4707	79.1
Refuse to answer/Don't know	50	1.0		9	3.0		7	1.8			2	1.4				68	1.1
Caregiver education			--			χ2(5)=8.11, p=.27, V=.04			χ2(5)=21.23, p<.001, V=.06	χ2(5)=27.74, p<.001, V=.20			χ2(5)=10.75, p=.15, V=.05	χ2(5)=4.74, p=.56, V=.10	χ2(5)=11.84, p=.12, V=.15		
< High school diploma	175	3.4		6	2.0		19	4.8			4	2.7				204	3.4
High school diploma/GED	481	9.4		22	7.4		41	10.4			7	4.8				551	9.3
Some college/Associate degrees	1345	26.3		93	31.2		136	34.6			49	33.3				1623	27.3
Bachelors	1539	30.1		90	30.2		100	25.4			52	35.4				1781	29.9
Postgraduate degrees	1456	28.5		84	28.2		93	23.7			34	23.1				1667	28.0
Refuse to answer/Don't know	114	2.2		3	1.0		4	1.0			1	0.7				122	2.1
Caregiver marital status			--			χ2(6)=10.77, p=.20, V=.04			χ2(6)=15.33, p=.07, V=.05	χ2(6)=5.82, p=.56, V=.09			χ2(6)=2.61, p=.85, V=.02	χ2(6)=4.66, p=.66, V=.10	χ2(6)=4.22, p=.71, V=.09		
Married	3697	72.3		200	67.1		263	66.9			104	70.7				4264	71.7
Widowed	34	0.7		5	1.7		6	1.5			0	0.0				45	0.8
Divorced	437	8.6		32	10.7		32	8.1			12	8.2				513	8.6
Separated	179	3.5		12	4.0		19	4.8			7	4.8				217	3.6
Never married	481	9.4		32	10.7		52	13.2			17	11.6				582	9.8
Living with partner	246	4.8		17	5.7		20	5.1			6	4.1				289	4.9
Refuse to answer/Don't know	36	0.7		0	0.0		1	0.3			1	0.7				38	0.6
Total combined family income (USD)			--			χ2(10)=14.87, p=.27, V=.05			χ2(10)=13.43, p=.33, V=.05	χ2(10)=39.98, p<.001, V=.24			χ2(10)=8.68, p=.65, V=.04	χ2(10)=17.47, p=.15, V=.20	χ2(10)=7.46, p=.72, V=.12		
Less than $5,000	124	2.4		7	2.3		12	3.1			5	3.4				148	2.5
$5,000 through $11,999	135	2.6		11	3.7		13	3.3			7	4.8				166	2.8
$12,000 through $15,999	108	2.1		4	1.3		11	2.8			4	2.7				127	2.1
$16,000 through $24,999	181	3.5		10	3.4		17	4.3			3	2.0				211	3.5
$25,000 through $34,999	263	5.1		10	3.4		29	7.4			10	6.8				312	5.2
$35,000 through $49,999	368	7.2		30	10.1		31	7.9			10	6.8				439	7.4
$50,000 through $74,999	617	12.1		32	10.7		54	13.7			20	13.6				723	12.2
$75,000 through $99,999	714	14.0		56	18.8		52	13.2			18	12.2				840	14.1
$100,000 through $199,999	1569	30.7		87	29.2		106	27.0			49	33.3				1811	30.4
$200,000 and greater	640	12.5		29	9.7		36	9.2			14	9.5				719	12.1
Refuse to answer/Don't know	391	7.7		22	7.4		32	8.1			7	4.8				452	7.6
Stop Signal reaction time, mean in ms	264.28 (64.66)	--	--	262.23 (76.80)	--	--	263.51 (72.97)	--	--		256.85 (68.57)	--	--			262.29 (65.98)	--

Notes. TD = typically-developing; ADHD = attention-deficit/hyperactivity disorder. The omnibus test of sex differences was to test overall differences in class proportions between boys and girls, which were largely driven by the ADHD groups.

Table S3. Neuroimaging variables for latent variable modelling

Domain	ABCD variable name	Region name
**Response Inhibition**	tfmri_sacsvcg_bcdk_smlh	L Supramarginal gyrus
(Correct stop vs. correct go contrast)	tfmri_sacsvcg_bcdk_ifpalh	L Inferior parietal cortex
	tfmri_sacsvcg_bcdk_laoclh	L Lateral occipital cortex
	tfmri_sacsvcg_bcdk_pstglh	L Pars triangularis
	tfmri_sacsvcg_bcdk_psobslh	L Pars orbitalis
	tfmri_sacsvcg_bcdk_laobofrlh	L Lateral orbital frontal cortex
**Error processing**	tfmri_saisvcg_bcdk_sufrrh	R Superior frontal gyrus
(Incorrect stop vs. correct go contrast)	tfmri_saisvcg_bcdk_rmdfrrh	R Rostral middle frontal gyrus
	tfmri_saisvcg_bcdk_ifparh	R Inferior parietal cortex
	tfmri_saisvcg_bcdk_smrh	R Supramarginal gyrus
	tfmri_saisvcg_bcdk_pstgrh	R Pars triangularis
	tfmri_saisvcg_bcdk_psoperh	R Pars opercularis
	tfmri_saisvcg_bcdk_laobofrrh	R Lateral orbital frontal cortex
	tfmri_saisvcg_bcdk_sufrlh	L Superior frontal gyrus
	tfmri_saisvcg_bcdk_racgelh	L rostral anterior cingulate
	tfmri_saisvcg_bcdk_sutprh	R superior temporal gyrus
	tfmri_saisvcg_bcdk_bktsrh	R Bank superior temporal sulcus
	tfmri_saisvcg_bcdk_pstglh	L Pars triangularis
	tfmri_saisvcg_bcdk_psobslh	L Pars orbitalis
	tfmri_saisvcg_bcdk_laobofrlh	L Lateral orbital frontal cortex
	tfmri_saisvcg_bcdk_cdmdfrrh	R Caudal middle frontal gyrus
	tfmri_saisvcg_bcdk_precnrh	R Precentral gyrus
	tfmri_saisvcg_bcdk_supalh	L superior parietal cortex
	tfmri_saisvcg_bcdk_laoclh	L Lateral occipital cortex
	tfmri_saisvcg_bcdk_smlh	L Supramarginal gyrus
	tfmri_saisvcg_bcdk_ifpalh	L Inferior parietal cortex
	tfmri_saisvcg_bcdk_suparh	R superior parietal cortex
	tfmri_saisvcg_bcdk_pcurh	R Precuneus cortex
	tfmri_saisvcg_bcdk_bktslh	L Bank superior temporal sulcus

Notes. L = left hemishpere; R = right hemisphere. The identified cortical regions showed significant test-retest reliability (ICCs > .60) in Korucuoglu et al. (6). No subcortical regions showed significant test-retest reliability in the study.

Table S4. Model fit information and measurement invariance of response inhibition using a priori selected regions

Response Inhibition - Model fit

$\chi 2$	df	p-value	CFI	TFI	RMSEA	RMSEA_LI	RMSEA_UI	SRMR
59.62	23	<.001	0.99	0.99	0.02	0.01	0.02	0.01

Response Inhibition - Measurement invariance testing

Model	CFI	ΔCFI	RMSEA	ΔRMSEA
Configural	1.00	--	0.03	--
Metric	1.00	0.00	0.02	-0.01
Scalar	1.00	0.00	0.02	0.00

Notes. Comparison based on criteria proposed by Chen (8): ΔCFI> .01 and ΔRMSEA>.015, reject invariance.

Table S5. Model fit information and measurement invariance of error processing using a priori selected regions

Error Processing - Model fit

$\chi 2$	df	p-value	CFI	TFI	RMSEA	RMSEA_LI	RMSEA_UI	SRMR
56.79	36	0.02	1.00	0.99	0.01	0.01	0.02	0.01

Error Processing - Measurement invariance testing

Model	CFI	ΔCFI	RMSEA	ΔRMSEA
Configural	0.99	--	0.02	--
Metric	0.99	-0.01	0.02	0.00
Scalar	0.99	0.00	0.02	0.00

Notes. Comparison based on criteria proposed by Chen (8): ΔCFI> .01 and ΔRMSEA>.015, reject invariance.

Table S6. Model fit information and measurement invariance of response inhibition using all available regions

Response Inhibition - Model fit

χ2	df	p-value	CFI	TFI	RMSEA	RMSEA_LI	RMSEA_UI	SRMR
49.27	29	0.01	1.00	1.00	0.01	0.01	0.02	0.01

Response Inhibition - Measurement invariance testing

Model	CFI	ΔCFI	RMSEA	ΔRMSEA
Configural	1.00	--	0.03	--
Metric	1.00	0.00	0.03	0.00
Scalar	1.00	0.00	0.03	0.00

Notes. Comparison based on criteria proposed by Chen (8): ΔCFI> .01 and ΔRMSEA>.015, reject invariance.

Table S7. Model fit information and measurement invariance of error processing using all available regions

Error Processing - Model fit

$\chi 2$	df	p-value	CFI	TFI	RMSEA	RMSEA_LI	RMSEA_UI	SRMR
114.24	22	<.001	0.99	0.99	0.03	0.02	0.03	0.01

Error Processing - Measurement invariance testing

Model	CFI	ΔCFI	RMSEA	ΔRMSEA
Configural	1.00	--	0.00	--
Metric	1.00	0.00	0.00	0.00
Scalar	1.00	0.00	0.00	0.00

Notes. Comparison based on criteria proposed by Chen (8): ΔCFI> .01 and ΔRMSEA>.015, reject invariance.

Table S8. Group comparison of latent brain activation pattern

	Group 1 intercept	Group 2 intercept	Group 2 - Group 1 estimate	SE	p	d
**Response Inhibition**						
TD vs ADHD-IRR	0.00	0.31	0.31	0.20	0.26	0.69
TD vs ADHD	0.00	-0.23	-0.23	0.17	0.28	0.56
TD vs IRR	0.00	-0.09	-0.09	0.21	0.66	0.20
ADHD-IRR vs ADHD	0.31	-0.23	-0.54	0.23	0.12	1.12
ADHD-IRR vs IRR	0.31	-0.09	-0.40	0.20	0.16	0.89
ADHD vs IRR	-0.23	-0.09	0.14	0.23	0.61	0.29
**Error Processing**						
TD vs ADHD-IRR	0.00	0.16	0.16	0.14	0.34	0.43
TD vs ADHD	0.00	-0.16	-0.16	0.13	0.32	0.45
TD vs IRR	0.00	-0.27	-0.27	0.16	0.20	0.69
ADHD-IRR vs ADHD	0.16	-0.16	-0.33	0.14	0.12	0.87
ADHD-IRR vs IRR	0.16	-0.27	-0.44	0.23	0.16	0.92
ADHD vs IRR	-0.16	-0.27	-0.11	0.19	0.61	0.26

Notes. TD = typically-developing; ADHD = attention-deficit hyper-reactivity disorder; IRR = irritability. Results were adjusted for child's age, sex, race, ethnicity, caregiver's education, caregiver's marital status, total family income (past 12 months), and scan site. Unstandardized estimates were shown.

Table S9. Sex by group comparison of individual region of interest

	Sum of square	Mean square	Num df	Den df	F	p	η^2
**Response Inhibition**							
L Inferior parietal cortex	0.01	0.00	3	5262	0.20	0.90	0.75
L Lateral occipital cortex	0.25	0.08	3	5260	1.94	0.40	0.75
L Pars orbitalis	0.14	0.05	3	5256	1.10	0.88	0.75
L Supramarginal gyrus	0.01	0.00	3	5260	0.23	0.90	0.75
**Error Processing I**							
L Lateral orbital frontal cortex	0.24	0.08	3	5262	0.76	0.90	0.75
L Pars orbitalis	0.23	0.08	3	5260	0.40	0.90	0.75
R Lateral orbital frontal cortex	0.09	0.03	3	5260	0.28	0.90	0.75
**Error Processing II**							
L Lateral occipital cortex	0.05	0.02	3	5259	0.29	0.90	0.75
L Superior parietal cortex	0.14	0.05	3	5259	2.16	0.40	0.75
R Superior parietal cortex	0.20	0.07	3	5258	2.77	0.40	0.75

Notes. L = left hemisphere; R = right hemisphere. Results were adjusted for child's age, race, ethnicity, caregiver's education, caregiver's marital status, total family income (past 12 months), and scan site.

Table S10. Model fit information and measurement invariance of response inhibition using a priori selected regions and family clustering

Response Inhibition - Model fit

$\chi 2$	df	p-value	CFI	TFI	RMSEA	RMSEA_LI	RMSEA_UI	SRMR
66.81	23	<.001	0.99	0.99	0.02	0.01	0.02	0.01

Response Inhibition - Measurement invariance testing

Model	CFI	ΔCFI	RMSEA	ΔRMSEA
Configural	1.00	--	0.03	--
Metric	1.00	0.00	0.02	-0.01
Scalar	1.00	0.00	0.02	0.00

Notes. Comparison based on criteria proposed by Chen (8): ΔCFI> .01 and ΔRMSEA>.015, reject invariance.

Table S11. Model fit information and measurement invariance of error processing using a priori selected regions and family clustering

Error Processing - Model fit

$\chi2$	df	p-value	CFI	TFI	RMSEA	RMSEA_LI	RMSEA_UI	SRMR
55.80	36	0.02	1.00	0.99	0.01	0.004	0.02	0.01

Error Processing - Measurement invariance testing

Model	CFI	ΔCFI	RMSEA	ΔRMSEA
Configural	0.99	--	0.02	--
Metric	0.99	-0.01	0.02	0.00
Scalar	0.99	0.00	0.02	0.00

Notes. Comparison based on criteria proposed by Chen (8): ΔCFI> .01 and ΔRMSEA>.015, reject invariance.

Table S12. Comparison of latent brain activation patterns across groups using a priori selected regions and family clustering

	Group 1 intercept	Group 2 intercept	Group 2 - Group 1 estimate	SE	p	d
**Response Inhibition**						
TD vs ADHD-IRR	0.00	0.17	0.17	0.16	0.71	0.43
TD vs ADHD	0.00	-0.17	-0.17	0.14	0.61	0.47
TD vs IRR	0.00	-0.56	-0.56	0.17	**0.01**	1.39
ADHD-IRR vs ADHD	0.17	-0.17	-0.34	0.20	0.41	0.76
ADHD-IRR vs IRR	0.17	-0.56	-0.73	0.23	**0.01**	1.55
ADHD vs IRR	-0.17	-0.56	-0.39	0.21	0.37	0.85
**Error Processing I**						
TD vs ADHD-IRR	0.00	-0.13	-0.13	0.24	0.90	0.26
TD vs ADHD	0.00	0.06	0.06	0.34	0.90	0.10
TD vs IRR	0.00	0.20	0.20	0.47	0.90	0.29
ADHD-IRR vs ADHD	-0.13	0.06	0.19	0.41	0.90	0.30
ADHD-IRR vs IRR	-0.13	0.20	0.33	0.52	0.90	0.45
ADHD vs IRR	0.06	0.20	0.14	0.57	0.90	0.18
**Error Processing II**						
TD vs ADHD-IRR	0.00	-0.06	-0.06	0.17	0.90	0.16
TD vs ADHD	0.00	-0.21	-0.21	0.14	0.49	0.55
TD vs IRR	0.00	-0.11	-0.11	0.27	0.90	0.20
ADHD-IRR vs ADHD	-0.06	-0.21	-0.14	0.21	0.90	0.31
ADHD-IRR vs IRR	-0.06	-0.11	-0.04	0.31	0.90	0.08
ADHD vs IRR	-0.21	-0.11	0.10	0.30	0.90	0.18

Notes. ADHD = attention-deficit/hyperactivity disorder; IRR = irritability; TD = typically developing. Results were adjusted for child's age, sex, race, ethnicity, caregiver's education, caregiver's marital status, total family income (past 12 months), and family clustering. Unstandardized estimates were shown.

Table S13. Group comparison of individual region of interest using family clustering

	Sum of square	Mean square	Num df	Den df	F	p	η^2
**Response Inhibition**							
L Inferior parietal cortex	0.07	0.02	3	5263	1.45	0.33	0.75
L Lateral occipital cortex	0.28	0.09	3	5265	2.31	0.18	0.76
L Pars orbitalis	0.24	0.08	3	5263	2.21	0.18	0.75
L Supramarginal gyrus	0.05	0.02	3	5259	1.14	0.41	0.71
**Error Processing I**							
L Lateral orbital frontal cortex	0.74	0.25	3	5265	2.35	0.18	0.75
L Pars orbitalis	2.53	0.84	3	5260	4.80	**0.02**	0.75
R Lateral orbital frontal cortex	1.03	0.34	3	5265	3.21	0.10	0.75
**Error Processing II**							
L Lateral occipital cortex	0.29	0.10	3	5258	1.95	0.20	0.74
L Superior parietal cortex	0.02	0.01	3	5250	0.36	0.78	0.67
R Superior parietal cortex	0.05	0.02	3	5261	0.73	0.59	0.71

Notes. L = left hemishpere; R = right hemishere. Results were adjusted for child's age, sex, race, ethnicity, caregiver's education, caregiver's marital status, total family income (past 12 months), and family clustering.

Table S14. Model fit information and measurement invariance of response inhibition using all available regions and family clustering

Response Inhibition - Model fit

χ2	df	p-value	CFI	TFI	RMSEA	RMSEA_LI	RMSEA_UI	SRMR
45.42	29	0.03	1.00	1.00	0.01	0.004	0.02	0.01

Response Inhibition - Measurement invariance testing

Model	CFI	ΔCFI	RMSEA	ΔRMSEA
Configural	1.00	--	0.03	--
Metric	1.00	0.00	0.03	0.00
Scalar	1.00	0.00	0.03	0.00

Notes. Comparison based on criteria proposed by Chen (8): ΔCFI> .01 and ΔRMSEA>.015, reject invariance.

Table S15. Model fit information and measurement invariance of error processing using all available regions and family clustering

Error Processing - Model fit

$\chi2$	df	p-value	CFI	TFI	RMSEA	RMSEA_LI	RMSEA_UI	SRMR
116.05	22	<.001	0.99	0.99	0.03	0.02	0.03	0.01

Error Processing - Measurement invariance testing

Model	CFI	ΔCFI	RMSEA	ΔRMSEA
Configural	1.00	--	0.00	--
Metric	1.00	0.00	0.00	0.00
Scalar	1.00	0.00	0.00	0.00

Notes. Comparison based on criteria proposed by Chen (8): ΔCFI> .01 and ΔRMSEA>.015, reject invariance.

Table S16. Group comparison of latent brain activation pattern using all available regions and family clustering

	Group 1 intercept	Group 2 intercept	Group 2 - Group 1 estimate	SE	p	d
**Response Inhibition**						
TD vs ADHD-IRR	0.00	0.31	0.31	0.20	0.24	0.69
TD vs ADHD	0.00	-0.23	-0.23	0.17	0.25	0.57
TD vs IRR	0.00	-0.09	-0.09	0.26	0.73	0.18
ADHD-IRR vs ADHD	0.31	-0.23	-0.54	0.25	0.13	1.07
ADHD-IRR vs IRR	0.31	-0.09	-0.40	0.32	0.28	0.70
ADHD vs IRR	-0.23	-0.09	0.14	0.30	0.70	0.26
**Error Processing**						
TD vs ADHD-IRR	0.00	0.16	0.16	0.11	0.24	0.49
TD vs ADHD	0.00	-0.16	-0.16	0.10	0.24	0.50
TD vs IRR	0.00	-0.27	-0.27	0.16	0.24	0.68
ADHD-IRR vs ADHD	0.16	-0.16	-0.33	0.15	0.13	0.85
ADHD-IRR vs IRR	0.16	-0.27	-0.44	0.19	0.13	1.00
ADHD vs IRR	-0.16	-0.27	-0.11	0.19	0.66	0.26

Notes. ADHD = attention-deficit/hyperactivity disorder; IRR = irritability; TD = typically developing. Results were adjusted for child's age, sex, race, ethnicity, caregiver's education, caregiver's marital status, total family income (past 12 months), and family clustering. Unstandardized estimates were shown.

Table S17. Comparison of latent brain activation patterns across groups using a priori selected regions with covariates removed

	Group 1 mean	Group 2 mean	Group 2 - Group 1 estimate	SE	p	d
**Response Inhibition**						
TD vs ADHD-IRR	0.00	0.02	0.02	0.01	0.18	0.18
TD vs ADHD	0.00	0.003	0.003	0.01	0.82	0.04
TD vs IRR	0.00	0.003	0.003	0.01	0.91	0.03
ADHD-IRR vs ADHD	0.02	0.003	-0.01	0.01	0.82	0.11
ADHD-IRR vs IRR	0.02	0.003	-0.01	0.01	0.82	0.10
ADHD vs IRR	0.003	0.003	-0.001	0.01	0.97	0.01
**Error Processing I**						
TD vs ADHD-IRR	0.00	0.05	0.05	0.02	**0.03**	0.40
TD vs ADHD	0.00	-0.02	-0.02	0.02	0.82	0.12
TD vs IRR	0.00	-0.02	-0.02	0.03	0.82	0.14
ADHD-IRR vs ADHD	0.05	-0.02	-0.07	0.02	**0.02**	0.49
ADHD-IRR vs IRR	0.05	-0.02	-0.08	0.04	0.18	0.40
ADHD vs IRR	-0.02	-0.02	-0.01	0.03	0.91	0.05
**Error Processing II**						
TD vs ADHD-IRR	0.00	0.003	0.003	0.01	0.91	0.03
TD vs ADHD	0.00	-0.01	-0.01	0.01	0.82	0.08
TD vs IRR	0.00	-0.01	-0.01	0.01	0.82	0.06
ADHD-IRR vs ADHD	0.003	-0.01	-0.01	0.01	0.82	0.07
ADHD-IRR vs IRR	0.003	-0.01	-0.01	0.01	0.82	0.08
ADHD vs IRR	-0.01	-0.01	0.00	0.01	0.97	0.00

Notes. ADHD = attention-deficit/hyperactivity disorder; IRR = irritability; TD = typically developing. Results were adjusted for scan site clustering. Unstandardized estimates were shown.

Table S18. Group comparison of latent brain activation pattern using all available regions with covariates removed

	Group 1 mean	Group 2 mean	Group 2 - Group 1 estimate	SE	p	d
**Response Inhibition**						
TD vs ADHD-IRR	0.00	0.01	0.01	0.01	0.90	0.08
TD vs ADHD	0.00	0.004	0.004	0.01	0.98	0.04
TD vs IRR	0.00	0.001	0.001	0.01	1.00	0.01
ADHD-IRR vs ADHD	0.01	0.004	-0.003	0.01	0.99	0.03
ADHD-IRR vs IRR	0.01	0.001	-0.01	0.02	0.99	0.05
ADHD vs IRR	0.004	0.001	-0.003	0.02	1.00	0.02
**Error Processing**						
TD vs ADHD-IRR	0.00	0.01	0.01	0.004	0.24	0.16
TD vs ADHD	0.00	0.00	0.00	0.01	1.00	0.00
TD vs IRR	0.00	-0.01	-0.01	0.01	0.53	0.11
ADHD-IRR vs ADHD	0.01	0.00	-0.01	0.01	0.53	0.11
ADHD-IRR vs IRR	0.01	-0.01	-0.02	0.01	0.24	0.20
ADHD vs IRR	0.00	-0.01	-0.01	0.01	0.53	0.11

Notes. TD = typically-developing; ADHD = attention-deficit hyper-reactivity disorder; IRR = irritability. Results were adjusted for scan site clustering. Unstandardized estimates were shown.
